# A *CARD9* Founder Mutation Disrupts NF-κB Signaling by Inhibiting BCL10 and MALT1 Recruitment and Signalosome Formation

**DOI:** 10.3389/fimmu.2018.02366

**Published:** 2018-10-31

**Authors:** Marieke De Bruyne, Levi Hoste, Delfien J. Bogaert, Lien Van den Bossche, Simon J. Tavernier, Eef Parthoens, Mélanie Migaud, Deborah Konopnicki, Jean Cyr Yombi, Bart N. Lambrecht, Sabine van Daele, Ana Karina Alves de Medeiros, Lieve Brochez, Rudi Beyaert, Elfride De Baere, Anne Puel, Jean-Laurent Casanova, Jean-Christophe Goffard, Savvas N. Savvides, Filomeen Haerynck, Jens Staal, Melissa Dullaers

**Affiliations:** ^1^Primary Immunodeficiency Research Lab, Department of Pulmonary Medicine, Centre for Primary Immunodeficiencies, Jeffrey Modell Diagnosis and Research Centre, Ghent University Hospital, Ghent, Belgium; ^2^Center for Medical Genetics, Ghent University and Ghent University Hospital, Ghent, Belgium; ^3^Department of Pediatric Immunology and Pulmonology, Centre for Primary Immunodeficiencies, Jeffrey Modell Diagnosis and Research Centre, Ghent University Hospital, Ghent, Belgium; ^4^Laboratory of Immunoregulation, VIB-UGent Center for Inflammation Research, Ghent, Belgium; ^5^Laboratory for Protein Biochemistry and Biomolecular Engineering, Department of Biochemistry and Microbiology, Ghent University, Ghent, Belgium; ^6^VIB-UGent Center for Inflammation Research, Ghent, Belgium; ^7^VIB Bioimaging Core, VIB, Ghent, Belgium; ^8^Laboratory of Human Genetics of Infectious Diseases, INSERM UMR1163, Necker Medical School, Imagine Institute, Paris Descartes University, Paris, France; ^9^Infectious Diseases Department, Saint-Pierre University Hospital, Université Libre de Bruxelles, Brussels, Belgium; ^10^Department of Internal Medicine and Infectious Diseases, Cliniques Universitaires Saint Luc, Université Catholique de Louvain, Brussels, Belgium; ^11^Department of Internal Medicine, Ghent University, Ghent, Belgium; ^12^Department of Dermatology, Ghent University Hospital, Ghent, Belgium; ^13^Unit of Molecular Signal Transduction in Inflammation, Department of Biomedical Molecular Biology, VIB-UGent Center for Inflammation Research, Ghent University, Ghent, Belgium; ^14^St Giles Laboratory of Human Genetics of Infectious Diseases, Rockefeller University, New York, NY, United States; Pediatric Hematology-Immunology Unit, Necker Hospital, New York, NY, United States; ^15^Immunodeficiency Unit, University Hospital ULB Erasme, Brussels, Belgium

**Keywords:** CARD9 deficiency, founder mutation, BCL10, MALT1, CBM complex, NF-κB, filament, signalosome

## Abstract

**Background:** Inherited CARD9 deficiency constitutes a primary immunodeficiency predisposing uniquely to chronic and invasive fungal infections. Certain mutations are shown to negatively impact CARD9 protein expression and/or NF-κB activation, but the underlying biochemical mechanism remains to be fully understood.

**Objectives:** To investigate a possible founder origin of a known CARD9 R70W mutation in five families of Turkish origin. To explore the biochemical mechanism of immunodeficiency by R70W CARD9.

**Methods:** We performed haplotype analysis using microsatellite markers and SNPs. We designed a model system exploiting a gain-of-function (GOF) CARD9 L213LI mutant that triggers constitutive NF-κB activation, analogous to an oncogenic CARD11 mutant, to study NF-κB signaling and signalosome formation. We performed reporter assays, immunoprecipitation and confocal imaging on HEK cells overexpressing different CARD9 variants.

**Results:** We identified a common haplotype, thus providing evidence for a common Turkish founder. CARD9 R70W failed to activate NF-κB and abrogated NF-κB activation by WT CARD9 and by GOF CARD9. Notably, R70W CARD9 also exerted negative effects on NF-κB activation by CARD10, CARD11, and CARD14. Consistent with the NF-κB results, the R70W mutation prevented GOF CARD9 to pull down the signalosome partner proteins BCL10 and MALT1. This reflected into drastic reduction of BCL10 filamentous assemblies in a cellular context. Indeed, structural analysis revealed that position R70 in CARD9 maps at the putative interface between successive CARD domains in CARD9 filaments.

**Conclusions:** The R70W mutation in CARD9 prevents NF-κB activation by inhibiting productive interactions with downstream BCL10 and MALT1, necessary for assembly of the filamentous CARD9-BCL10-MALT1 signalosome.

## Introduction

In the past decade, the importance of IL-17-mediated immunity in host defense against fungal infections has become clear, in large part by studies of patients with inborn errors of IL-17 immunity and relentless chronic mucocutaneous candidiasis (CMC) as a common theme. Mutations in key components of the IL-17A/IL-17F pathway were shown to compromise cellular responses to these cytokines, thus predisposing to CMC (1–3). Furthermore, patients with inherited disorders affecting IL-17A/IL-17F production (low Th17 cells) often present CMC in combination with other infections/clinical features (1–3). Caspase recruitment domain family member 9 (CARD9) is an adaptor molecule connecting dectin-1 fungal sensing to nuclear factor κB (NF-κB) signaling and thus important in host defense against fungal infections. It was first shown that Card9-deficient (Card9^−/−^) mice are highly susceptible to fungal infection (*Candida albicans*) due to their inability to produce proinflammatory cytokines in response to dectin-1 stimulation e.g. by zymosan or *C. albicans* (4). Subsequently in 2009, autosomal recessive (AR) *CARD9* deficiency was identified to be responsible for recurrent superficial fungal infections and central invasion with *Candida* spp. (5).

To date, 63 patients of 38 kindreds originating from at least 14 countries with *CARD9* mutations have been reported (see Supplemental Table 1, including references). Even though clinical presentation is highly variable, CARD9 deficiency predisposes uniquely to chronic and invasive fungal infections in otherwise healthy individuals (6). As proposed in a recent review (7), clinical presentation can be classified as (1) isolated CMC, (2) infections of skin and subcutaneous tissues with fungal species including *Trichopython* spp, *Phialophora verrucosa* and *Corynespora cassiicola*, and (3) systemic fungal disease manifesting primarily as meningoencephalitis, osteomyelitis and intra-abdominal infections with *Candida* spp, *Trichophyton* spp*, Exophiala spp*, and rarely *Aspergillus* (5–10). All patients are homozygous or compound heterozygous for 21 different mutations in the CARD9 CARD and coiled-coil (CC) domains (**Figure 2A**, Supplemental Table 1). Most of these mutations are missense and nonsense mutations, but also frameshifts, synonymous substitutions and an in-frame deletion have been described.

CARD9 is an intracellular adaptor molecule that mediates signaling downstream of C-type lectin receptors, including Dectin-1 and Dectin-2, upon fungal recognition by myeloid or epithelial cells. CARD9 forms signaling complexes with B-cell CLL/lymphoma 10 (BCL10) and mucosa-associated lymphoid tissue lymphoma translocation protein 1 (MALT1), termed CARD-CC/BCL10/MALT1 (CBM) complexes. Such CBM signalosomes mediate NF-κB activation resulting in induction of pro-inflammatory cytokines, such as IL-1, IL-6, and IL-23. These promote the differentiation of T lymphocytes into IL-17 producing T cells, further mediating anti-fungal immunity (7).

Caspase recruitment domains (CARD) are the common motif in the CARD subfamily of death domain proteins containing 33 human CARD-containing proteins. Homotypic CARD-CARD domain interactions between CARD-proteins regulate the assembly of many signaling complexes, including the CBM signalosome and many others such as the apoptosome and the inflammasome (11). CARD9 belongs to a phylogenetically distinct family of CARD-proteins along with CARD10 (CARMA3), CARD11 (CARMA1), and CARD14 (CARMA2) which are defined by their CARD and coiled-coil (CC) domains (11, 12). The four different CARD-CC proteins all form CBM signaling complexes, mediated mainly by CARD-domain hetero-multimerization. CBM complexes form a critical link between cell-surface receptors that associate with extracellular molecular antigens and downstream activation of NF-κB. Active CBM signalosomes are formed when pre-existing filamentous BCL10 structures are nucleated by activation of CARD-CC proteins (13–15). Genetic defects in several components of the CBM complex have been linked to human immunopathology. Somatic and germline gain-of-function (GOF) mutations in *CARD11, MALT1*, and *BCL10* cause B cell lymphomas and lymphoproliferative disorders (16). Biallelic loss-of-function (LOF) mutations in *CARD11, MALT1*, and *BCL10* lead to combined immunodeficiencies (16–18) whereas the here-discussed CARD9 deficiency predisposes exclusively to fungal infections.

The R70W (c.208C>T) *CARD9* mutation was previously reported in four patients of three unrelated families of consanguineous Turkish decent, living in Belgium or France (10, 19). We here expand the R70W CARD9 cohort to 11 patients of five unrelated families all from Turkish descent and we provide evidence supporting a common founder origin of this mutation. The R70W *CARD9* variant was shown to affect CARD9 protein expression and is associated with impaired NF-κB activation (10). In this study, we interrogated the biochemical mechanism of the R70W mutation in CARD9 and found that it disrupts NF-κB activation by abrogating recruitment of BCL10 and subsequent filamentous signalosome formation.

## Materials and methods

### Patients

Patients were recruited in Ghent University Hospital, Ghent, Belgium (19); University Hospital ULB Erasme, Brussels, Belgium; Saint Pierre University hospital, ULB, Brussels, Belgium; University Hospital Saint Luc, UCL, Brussels, Belgium; the Infectious Diseases Unit in Bretonneau Hospital, Tours, France and Necker Hospital, Paris, France (10). This study was conducted in accordance with the Helsinki Declaration and was approved by the ethical committee of Ghent University Hospital (2012/593). All patients and their relatives provided written informed consent for participation in the study.

Extended case reports are provided in the online supplement.

### T-cell proliferation

T-cell proliferation assay was a standard lymphocyte proliferation test (LTT) performed in routine immunology laboratories. Responses to *C. albicans* and the mitogens phytohemaglutinin (PHA), Concanavalin A (ConA), or Pokeweed mitogen (PWM) were assessed.

### Immunologic assessment of Th17 function and fungal recognition

For intracellular cytokine staining, 1^*^10^6^ PBMC were cultured in complete medium with phorbol 12-myristate 13-acetate (PMA) (200 ng/mL) and Ionomycin (1 μg/mL) in the presence of Brefeldin A (20 μg/mL) (all from Sigma). Cells were stained with CD4 T cell surface markers (CD3, CD4, CD45RO), fixed and then stained intracellularly for IL-17A (eBio64dec17) and IFNg (4s.B3, eBioscience). IL-17+ cells were scored within CD3+CD4+CD45RO+ cells. In addition, IL-17 production upon stimulation with SEB or PMA/ionomycin was measured by ELISA.

To test fungal recognition, 2^*^10^5^ PBMC were cultured for 2 days in complete medium without or with heat-killed *Candida albicans* (Invivogen, 10 μg/mL). ELISA for IL-6 was performed on supernatants (eBioscience Ready-Set-Go).

### Haplotype analysis

Genomic DNA was isolated from whole blood cells according to standard procedures. Identity-by-descent (IBD) mapping was carried out in two affected individuals (F2 III:2, III:3) from family 2 by genome-wide single-nucleotide polymorphism (SNP) arrays using the HumanCytoSNP-12 BeadChip platform (Illumina, San Diego, CA). Ten IBD regions (>1 Mb) were identified using PLINK software (20). Starting from the IBD region (3.3 Mb) encompassing CARD9, four microsatellite markers were selected using NCBI Map Viewer (including genetic maps deCODE, Généthon, Marshfield and Rutgers Map v.3). In addition, five tagging SNP markers (UCSC Table Browser) were genotyped to further delineate the common haplotype. Primers were designed with Primer3Plus. Fragment analysis and sequencing were performed on the ABI 3730XL DNA Analyzer (Applied Biosystems). Data analysis of the microsatellite markers was performed with the GeneMapper v5 software (Applied Biosystems) and SNP markers were analyzed with SeqScape v2.5 (Life Technologies). Microsatellite and SNP markers were genotyped for haplotype analysis in ten affected patients and eleven healthy family members of the five families.

### Cloning and mutagenesis

Plasmids of the cloned genes were deposited in the LMBP/GeneCorner plasmid collection for public access along with detailed descriptions of cloning strategy and plasmid sequence (http://www.genecorner.ugent.be). Identification of the conserved site corresponding to the oncogenic CARMA1 L225LI (L232LI in long splice form) mutation was done in a MUSCLE multiple sequence alignment of a wide selection of CARD-CC proteins from Cnidaria to humans using UGENE (21). A pcDNA3-Flag (LMBP: 9609) or pENTR3C (LMBP: 9877) clone of human CARD9 was used as a template to generate the R70W (LMBP: 10266), L213LI (LMBP: 10178) and the R70W/L213LI (LMBP: 10265) mutants by PCR with Universe High-Fidelity Hot Start Taq (Bimake).

Two truncated CARD9 variants were generated. One downstream of the first CC domain CARD9-Q295X (LMBP: 10532) found in 11 patients and one downstream of the second CC domain CARD9-E419X (LMBP: 10457) constructed based on the last residue of the CC domain.

To test the effect of R70W CARD9 on the other CARD-CC proteins, analogously auto-actived mutants were generated by introducing a premature stop codon in between the CC-domain and the downstream C terminal inhibitory domain: CARD9-E419X (LMBP: 10457), CARD10-ΔC (LMBP: 10459), CARD11-ΔC (LMBP: 10458), and CARD14-ΔC (LMBP: 10460).

### Cell culture, transfection, and expression analysis

MALT1 deficient HEK293T cells (generated in house, clone #36) (22) were grown under standard conditions (DMEM, 10% FCS, 5% CO_2_, 37°C) and transfected with the calcium phosphate method (23). For NF-κB reporter assays, the indicated CARD9 construct was co-transfected with an NF-κB-dependent luciferase reporter expression plasmid (LMBP: 3249) and an actin promoter-driven β-galactosidase expression plasmid (LMBP: 4341) as transfection control. The cells were washed with PBS and lysed in luciferase lysis buffer (25 mM Tris pH7.8, 2 mM DTT, 2 mM CDTA, 10% glycerol, 1% Triton X-100). For the colorimetric determination (at 595 nm) of β-galactosidase activity, chlorophenol red-β-D-galactopyranoside (CPRG) (Roche diagnostics) was used as a substrate. Luciferase activity was measured by using beetle luciferin (Promega) as a substrate and luminescence was measured with the GloMax® 96 Microplate Luminometer (Promega). Luciferase data processing was done in LibreOffice (www.libreoffice.org) Calc. For evaluation of the MALT1-dependent signaling effects, the CARD9 clones were co-transfected with the NF-κB luciferase reporter and β-galactosidase expression plasmids in the MALT1 deficient HEK293T cells with or without reconstitution with human MALT1 (LMBP: 5536). For immunoprecipitation experiments, E-tagged human BCL10 (LMBP: 9637) and human MALT1 were co-transfected with the different CARD9 clones. Cells expressing BCL10 and MALT1 but no Flag-tagged CARD9 construct were used as background control for the immunoprecipitation. Immunoprecipitation was performed with anti-Flag antibody (F-3165, Sigma) in IP buffer (25 mM HEPES pH 7.5, 150 mM NaCl, 0.2% NP-40, 10% glycerol) with protein G magnetic Dynabeads (Invitrogen). For immunoblot detection, MALT1 was detected by a rabbit monoclonal antibody (EP603Y, Abcam), BCL10 with rabbit anti-E-tag antibody (ab66152, Abcam) and CARD9 with mouse anti-Flag antibody (F-3165, Sigma) or anti-Flag-HRP (A-8592, Sigma). All western blots were developed on an Odyssey scanner (LI-COR) except for HRP, which was developed with an Amersham Imager 600 (GE). For studies of inducible interactions of WT CARD9 with BCL10 and MALT1, transfected HEK293T cells were treated with 200 ng/ml PMA for 30 minutes before lysis and immunoprecipitation.

### Structural analysis of CARD9 and structural context of R70W CARD9

A structural model for human CARD9 was initially created from structure-based sequence alignments of the sequence encoding the CARD domain of human CARD9 (uniprot Q9H257) and was further improved via models computed via I-TASSER and QUARK (24). The homology model of the CARD domain of CARD9 was docked into a three-dimensional model representing the human BCL10 filament (Electron Microscopy Data Base code EMD-5729; (15) using the segment fitting algorithms implemented in Chimera (25).

### Confocal imaging

HEK293T cells were seeded in 8-well chamber slides (Ibidi). Ninety percent confluent cells were transfected using polyethylenimine [PEI, 25kDa branched (26)] with Flag-CARD9 and E-BCL10 (150 ng plasmid DNA each per well). The next day, cells were fixed with 4% PFA and were stained with mouse anti-Flag-tag (Sigma F3165) and rabbit anti-E-tag (Abcam ab66152) in triton containing staining buffer. Primary antibodies were detected using donkey anti-mouse-AF594 and donkey anti-rabbit 650.

Confocal images were captured with a Zeiss LSM880 confocal microscope (Zeiss, Zaventem, Belgium). Images were taken using a 63 × Pln Apo/1.4 oil objective. The pinhole was set at 1Airy Unit and scans ware made with a pixel dwell time of 2.62 μs. The scan area covered 800 by 800 pixels. Combined with a zoom of 2.2 this resulted in a pixel size of 0.077 μm. A Z-stack of 40–50 slices was recorded with a z-interval of 0.6 μm. 3D reconstructions were made in the 3D opacity mode of Volocity 6.3 (Perkin Elmer).

### Statistical analysis

Graphpad Prism was used to perform one-way ANOVA analysis followed by Tukey's *post-hoc* testing to assess statistical significance of the reporter assay data.

## Results

### Clinical presentation is highly variable in the R70W CARD9 patient cohort

**Figure 1:**
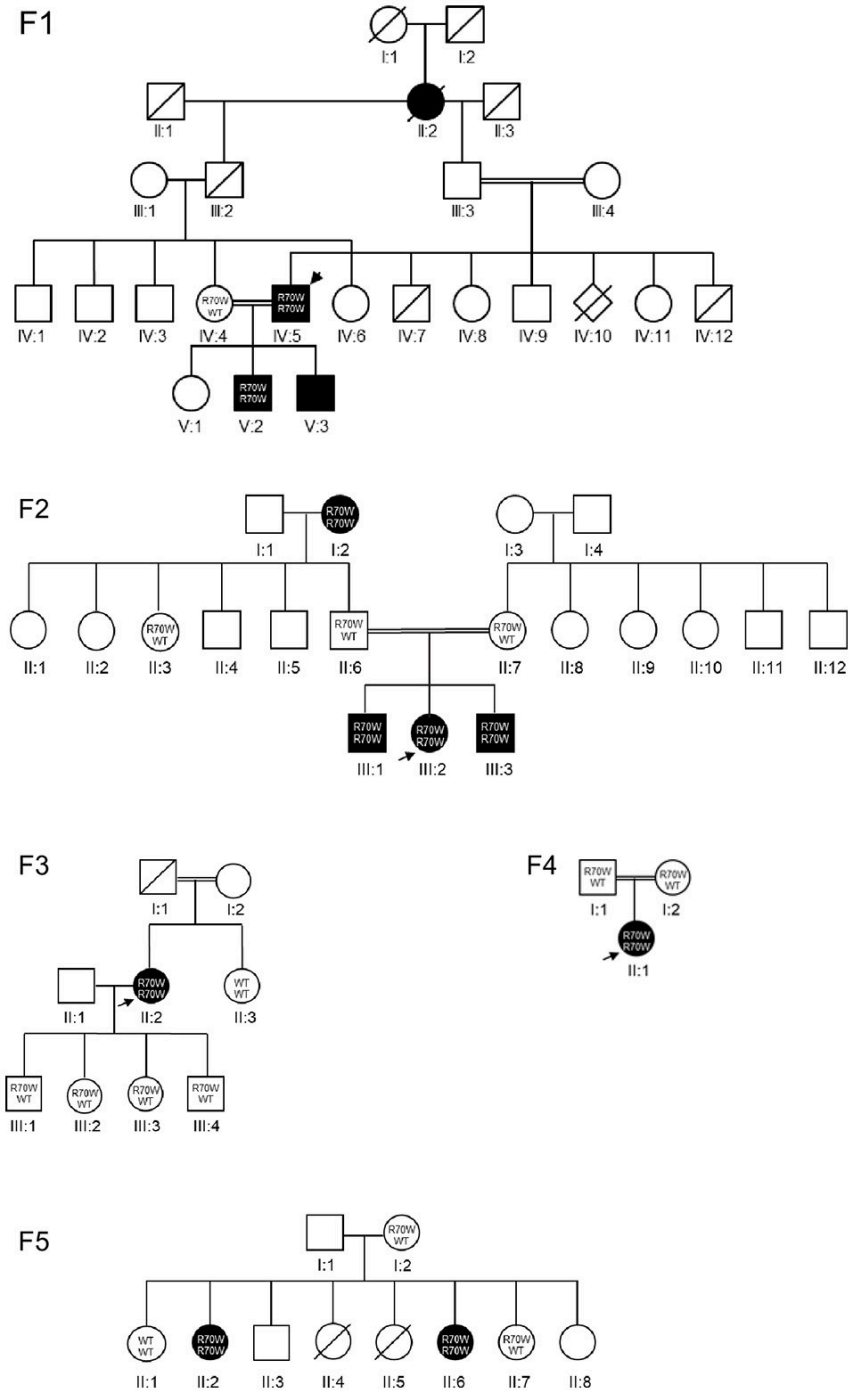
Pedigrees. *CARD9* R70W mutations were identified in five families. Each kindred is identified by a number (F1–F5), each generation by a Roman numeral (I–V), and each subject by an Arabic numeral (1–12). Square, circle and diamond shapes indicate male, female, and sex unknown, respectively. A double line represents reported consanguinity. Diagonal lines indicate deceased individuals. Filled symbols represent affected individuals, clear symbols represent unaffected individuals. The index patients are indicated with arrows. Where available, the genotype is mentioned. WT, wild-type allele.

**Figure 2:**
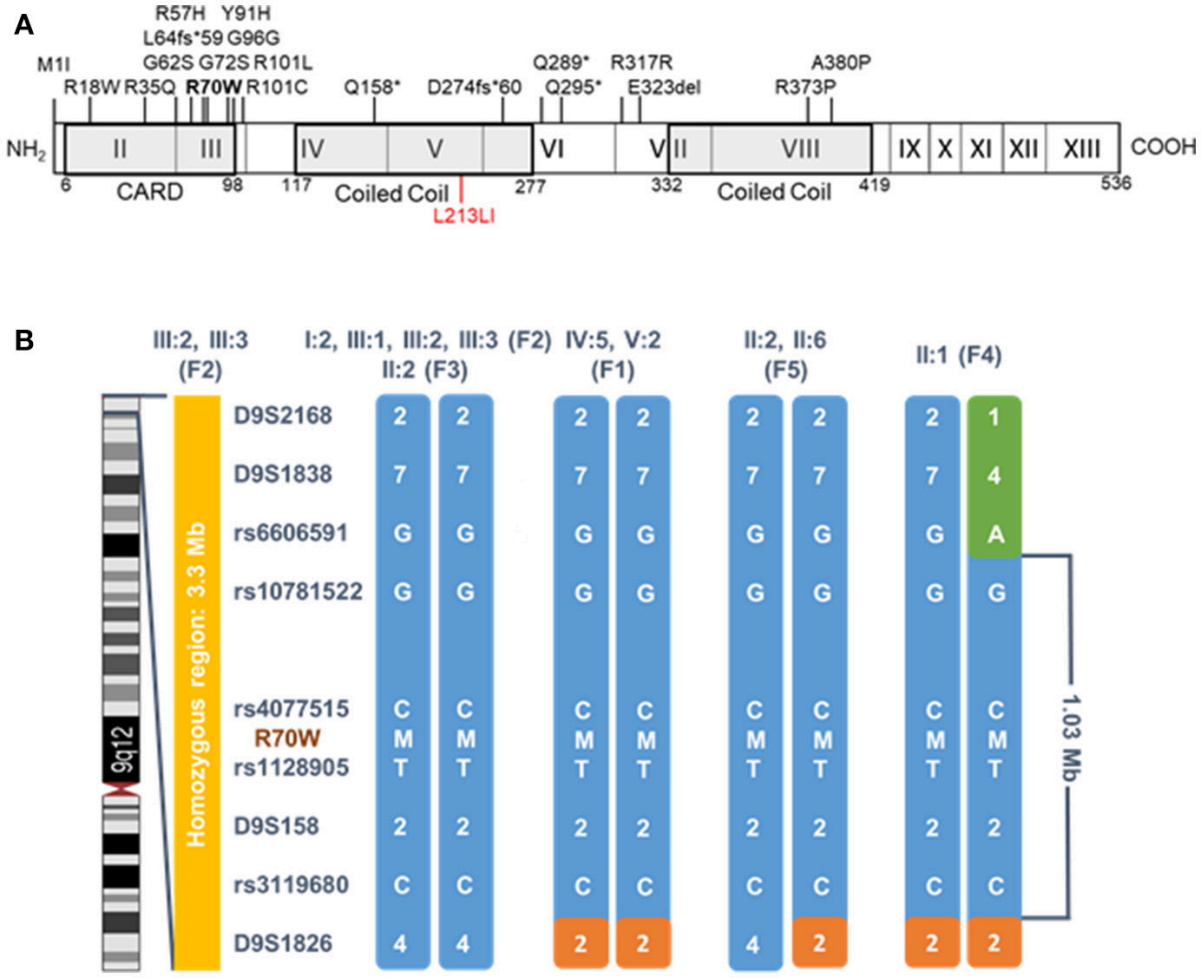
*CARD9* structure and haplotype analysis of the R70W (c.208C>T) mutation. **(A)** Schematic *CARD9* gene structure and reported disease-associated mutations. Roman numbered boxes represent exons. Gray boxes situate the CARD and Coiled Coil domains at protein level. The R70W mutation is shown in bold, the gain-of-function L213LI mutation in red. **(B)** Segregation analysis of flanking microsatellites and single nucleotide polymorphisms revealed a common haplotype of 1.03 Mb. Patients codes refer to the pedigrees in Figure 1. Segregation of the haplotype in all available family members is shown in Supplemental Figure 1. M, mutant allele; F, family.

We studied a cohort of 11 patients all harboring a known missense mutation in *CARD9*: R70W (c.208C>T). Family trees are provided in Figure 1. Because all patients originated from the Turkish provinces Afyonkarahisar and Eskişehir, we suspected a founder effect. Indeed, haplotype analysis provided evidence for a common Turkish founder (Figure 2B, Supplemental Figure 1).

Despite all bearing the R70W CARD9 mutation, the 11 patients present various fungal infections, including isolated CMC, chronic and invasive dermatophytosis, *Candida* meningoencephalitis and osteomyelitis, and intra-abdominal infections with *C. albicans* and *Aspergillus* spp (Table 1). Extra-pulmonary aspergillosis was only recently associated with AR CARD9 deficiency in 2 children (9). We here present an additional patient, F2 I.2, who reported to be healthy until she presented with retro-peritoneal aspergillosis at age 48. Consistent with previous reports (7, 10), the severity of the phenotype increases with age in the R70W CARD9 cohort, with childhood CMC often pre-dating invasive infections. This was also the case for patient F3 II.2, who developed CMC at age 36 and *C. albicans* encephalitis at age 39.

**Table 1:** Clinical and immunological details of the patients.

**Kindred**	**F1**			**F2**				**F3**	**F4**	**F5**		**Summary**
**Patient**	**IV:5 (index)**	**V:2**	**V:3**	**I:2**	**III:1**	**III:2 (index)**	**III:3**	**II:2 (index)**	**II:1 (index)**	**II:2 (index)**	**II:6**
Age of onset (y)	8	8	5	48	18	7	2.5	39	5	8	35	2.5–48
Age at last follow-up (y)	57	34	NA	59	18	16	10	42	8	43	37	8–59
Current age	57	35	32	65	20	17	11	46	15	NA	NA	11–65
Region of origin (Turkey)	Eskişehir	Eskişehir	Eskişehir	Emirdag	Emirdag	Emirdag	Emirdag	Afyon	NA	Emirdag	Emirdag	Afyon + Eskişehir + Emirdag
Country of living	Belgium	Belgium	Belgium	Belgium	Belgium	Belgium	Belgium	France	Belgium	Belgium	Belgium	Belgium + France
Associated fungal disease(s)	*Mucosal;* CMC, *systemic;* lymphadenitis, chronic + invasive dermatophytosis	*Mucosal;* CMC	*Mucosal;* CMC, *systemic;* encephalitis	*Systemic;* Retroperitoneal aspergillosis	*Mucosal;* Pityriasis versicolor	*Mucosal;* CMC	*Mucosal;* CMC	*Mucosal;* CMC, *systemic;* meningo-encephalitis	*Mucosal;* CMC, *systemic;* meningo-encephalitis	*Mucosal;* CMC, *systemic;* osteomyelitis	*Mucosal*; CMC, *systemic;* abdominal candidiasis, lymphadenitis	range
Non infectious disease(s)	nasal obstruction, allergic asthma, dyspepsia, gonarthrosis, caries	substance abuse, psychosis	hypopara-thyroidism	hypothyroidism, diabetes, osteoporosis		reccurent warts on foot soles	reccurent warts on foot soles, visual problems, headaches					auto-immune endocrinopathy, various
Fungus cultured	*Trichophyton violaceum, T. verrucosum, T. rubrum, Malassezia furfur*	NA	*C. albicans*	*Aspergillus fumigatus*	*T. rubrum*	*C. albicans*	*C. albicans*	*C. albicans*	*C. albicans*	*C. albicans*	*C. albicans*	*C. albicans, Trychophyton* spp*, Aspergillus*
CD4T	Normal	High	Normal	Normal	NA	Normal	Normal	Normal	NA	Normal	Normal	8/9 normal
CD8T	Normal	Normal	Normal	Normal	NA	Normal	Normal	Normal	NA	Normal	Normal	9/9 normal
LTT^$^	Mitogens^a, c, d^: normal^f^ Candida^a^: defect^h^	Mitogens^a, c, d^: normal^f^ Candida^a^: defect^h^	Mitogens^a, c, d^: normal^f^ Candida^a^: defect^h^	Mitogens^a, c, d^: normal^f^ Candida^a^: NA	NA	Mitogens^a, c, d^: strong^i^ Candida^a^: weak^g^	Mitogens^a, c, d^: strong^i^ Candida^a^: weak^g^	Mitogens^a, c, d^: normal^f^ Candida^a^: normal^f^	NA	Mitogens^b, c, e^: strong^i^ Candida: NA	Mitogens^b, c, f^: normal^a^ Candida: NA	5/8 decreased to *Candida*
Bc	Normal	Normal	Normal	NA	NA	Normal	Normal	Low	NA	Normal	Normal	7/8 normal
NKc	Normal	Normal	Normal	Normal	NA	Normal	Normal	Normal	NA	Normal	Normal	9/9 normal
IgE^£^	High^l^ (18,200 kU/L) Ref: 0-100 kU/L	High^l^ (843 kU/L) Ref: 0-100 kU/L	Increased^k^ (161 kU/L) Ref: 0-100 kU/L	Increased^k^(276.7 kU/L) Ref: 0-100 kU/L	NA	Normal^j^ (20.5 kU/L) Ref: 0-200 kU/L	Normal^j^ (20.6 kU/L) Ref: 0-90 kU/L	NA	NA	Normal^j^ (114 kU/L) Ref: 0-200 kU/L	High^l^ (1,137 kU/L) Ref: 0-200 kU/L	5/8 hyper IgE
IgA	Normal (3.3 g/L) Ref: 0.83-4.07 g/L	Normal (4.1 g/L) Ref: 0.83-4.07 g/L	Normal (1.99 g/L) Ref: 0.83-4.07 g/L	Normal	NA	Normal (1.4 g/L) Ref:0.71-3.65 g/L	Normal (1.1 g/L) Ref:0.5-1.66 g/L	Normal	NA	Normal	Normal	8/8 normal
IgM	Normal (0.7 g/L) Ref: 0.34-2.14 g/L	Normal (0.7 g/L) Ref: 0.34-2.14 g/L	Normal (1.75 g/L) Ref: 0.34-2.14 g/L	Normal	NA	Normal (2.0 g/L) Ref: 0.40-2.48 g/L	Normal (0.9 g/L) Ref: 0.27-0.74 g/L	Normal	NA	Normal	Normal	9/9 normal
IgG	Normal (13.5 g/L) Ref: 7.0-16.0 g/L	Normal (13.9 g/L) Ref: 7.0-16.0 g/L	Normal (16.63 g/L) Ref: 7.0-16.0 g/L	Normal	NA	Normal (15.5 g/L) Ref: 7.0-16.0 g/L	Normal (11.9 g/L) Ref: 4.7-11.9 g/L	Normal	NA	Normal	Normal	9/9 normal
Eosinophilia	Yes	No	Yes	No	NA	No	No	Yes	Yes	No	No	4/10 eosinophilia
Th17 function^#^	ICS: Low^p^ ELISA^m^: Low^p^	ICS: Low^p^ ELISA^m^: Low^p^	NA	ICS: Low^p^ ELISA^m^: Low^p^	ICS: Normal° ELISA^m^: Normal°	ICS: Low^p^ ELISA^m^: Low^p^	ICS: Low^p^ ELISA^m^: Low^p^	ICS: Normal° ELISA^n^: Normal°	ICS: Normal° ELISA^n^: Normal°	ICS: Low^p^ ELISA^m, n^: Low^p^	ICS: Low^p^ ELISA^m, n^: Low^p^	7/10 decreased
Fungal recognition^§^ (IL-6 upon candida)	Low^s^	Low^s^	NA	Low-normal^r^	Normal^q^	Low^s^	Low^s^	Low-normal^r^	Low-normal^r^	NA	NA	6/8 decreased

*CMC, chronic mucocutaneous candidiasis; LTT, lymphocyte transformation test; NA, not available*.

*If available, reference values are mentioned for tests performed in a routine diagnostic setting. For research assays, no reference values are available. Results were scored compared to internal assay healthy controls (HC)*.

$ *For LTT the reference ranges depends on the lab where tests were run. The response on different stimuli was determined for 3 reference HCs to define internal criteria that can be used as cutoff. In addition the reference range was developed for fresh and frozen samples to take into account biological variability. To correct for interrun variability, the 3 reference HCs together with one variable HC were run together with the patient samples^(a)^. In another center a standard reference range was used^(b)^. Responses to C. albicans and the mitogens PHA^(c)^, ConA^(d)^ or PWM^(e)^ were assessed. LTT was scored normal^(f)^ if response comparable to HC, weak^(g)^ for values below reference range, defect^(h)^ for absent response and strong^(i)^ for stronger response compared to HC*.

£ *IgE was scored normal^(j)^ for values within the normal reference range, increased^(k)^ for values above the reference range and high^(l)^ for strong increased values. ^#^Th17 function was assessed by intracellular cytokine staining (ICS) (proportion IL-17+ cells after stimulation with PMA/ionomycin measured by flow cytometry) and measurement of IL-17 production upon stimulation with SEB^(m)^ and/or PMA/ionomycin^(n)^ by ELISA. Th17 function was scored normal^(o)^ for values within the HC range and low^(p)^ if strongly impaired in comparison with the HCs*.

§ *Fungal recognition (IL-6 secretion upon Candida) was scored normal^(q)^ for values within the HC range, low-normal^(r)^ for values below the HC range and low^(s)^ if secretion was strongly impaired*.

Routine immunological work up revealed generally normal T and B cell subsets in all patients (Table 1). Lymphocyte proliferation upon stimulation with *C*. *albicans* was decreased in 5 out of 8 patients tested. Five patients displayed elevated serum IgE levels and 4 had eosinophilia. Th17 function was significantly decreased in 7 out of 10 patients tested. Pro-inflammatory cytokine production upon *Candida* stimulation was decreased in 6 out of 8 patients available for testing. Neither of the parameters reflecting anti-fungal immunity (Th17, anti-*candida* response) correlated with disease severity. Detailed case reports can be found in the online supplement.

### R70W CARD9 does not activate NF-κB and inhibits NF-κB activation by WT CARD9 and a gain-of-function CARD9 mutant

**Figure 3:**
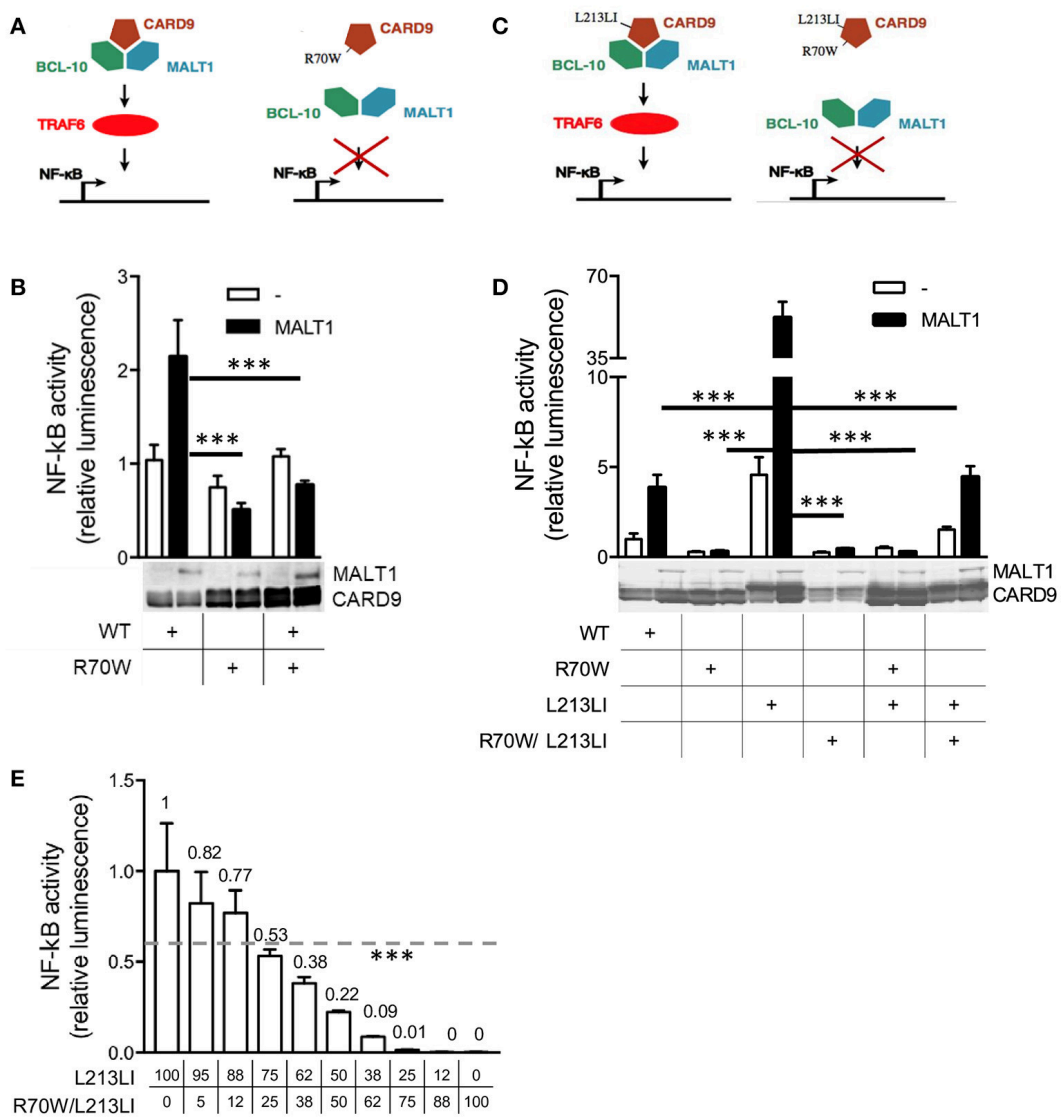
R70W CARD9 inhibits NF-κB transcriptional activity. **(A, C)** Schematic summaries of the experimental conditions in **(B,D)** respectively, adjusted from (13). **(B, D)** MALT1-deficient HEK293T cells were transfected with WT, R70W, L213LI and R70W/L213LI CARD9 as indicated, with fixed total DNA quantities, with or without MALT1 as indicated, together with an NF-κB-dependent luciferase reporter expression plasmid and a constitutively expressed β-galactosidase reporter gene. Luciferase values were normalized against β-galactosidase and expressed as fold induction compared to WT CARD9 without MALT1. Expression of MALT1 and CARD9 protein measured by western blot are shown below the graphs. **(E)** WT HEK293T cells were co-transfected with decreasing doses of L213LI DNA and increasing doses of R70W/L213LI CARD9 DNA (ng/well, doses specified under graph). The total DNA amount was kept constant at 400ng/well. Luciferase values were expressed as fold induction compared to L213LI CARD9 alone. All values under the dotted line are significant.Results shown in **(B,D,E)** are mean +/- standard deviation of 4 replicates. One out of 2–3 representative experiments is shown. Statistical analysis was performed on reporter assay data with one-way ANOVA and Tukey's multiple comparison's **(B, D, E)** post-testing. The most relevant statistical differences are shown, a list of all p-values is provided in Supplemental Tables 2–4. *p* < 0.001 (^***^) in all panels, in **(B,D)** only for reporter assays with MALT1 reconstitution.

Upon overexpression, wild-type (WT) CARD9 only moderately induced NF-κB activity in MALT-1-deficient HEK293T cells, but this was promoted by MALT-1 co-expression (27). Indeed, overexpression of WT CARD9 induced a 2- to 4-fold increase of MALT-1-dependent NF-κB reporter activity (Figures 3A,B,D). The R70W mutant, however, did not exhibit MALT-1-dependent NF-κB activation. More importantly, upon co-transfection of WT and R70W, the R70W mutant exerted an inhibitory effect on MALT-1-dependent NF-κB activation induced by WT CARD9 (Figures 3A,B). Off note, protein expression of the R70W allele was often weaker than the WT CARD9 allele, as had been reported before (10). We here report only data from those experiments where protein expression of R70W CARD9 was comparable to WT.

In that earlier report studying R70W CARD9 (10), co-expression of upstream (Dectin-1, Syk) and downstream (BCL10) signaling proteins together with fungal stimulation was used to determine the effects of CARD9 mutations on NF-κB activation. In that system of upstream activation, the R70W mutant also exhibited impaired NF-κB signaling. This strategy may also activate endogenous CARD10 present in HEK293T cells due to the high sequence conservation of the CARD-domain, which could mask effects of CARD9 mutations. To avoid this potential confounding factor and to specifically study signaling events downstream of CARD9, we generated a gain-of-function (GOF) mutant analogous to an oncogenic constitutively active CARD11 L225LI mutant. Like other oncogenic mutations in CARD11, this mutation is situated in the CC-domain and triggers constitutive NF-κB activation (17). The isoleucine insertion site is highly conserved ever since the single ancestral CARD-CC protein in the last common vertebrate ancestor (Supplemental Figure 2) and allowed us to generate an analogous isoleucine insertion mutant in CARD9 (L213LI). This CARD9 GOF mutant shows dramatically higher induction of NF-κB activity compared to WT, which we used as a model system for CARD9 activation, to test the effect of the R70W mutation. The L213LI CARD9 constitutive NF-κB activation was completely suppressed by the R70W mutation both in co-transfection of single mutants and in a double mutant setup (L213LI/R70W) (Figures 3C,D). Upon co-transfection, the double mutant also substantially reduced NF-κB activation by the GOF mutant but not as dramatically as the R70W single mutant. Since the protein expression of the R70W/L213LI double mutant was significantly lower than the other CARD9 alleles despite equal DNA dosage, this indicated a dose-dependency of the R70W inhibiting effect. Indeed, a cross-over dose-titration of the GOF L213LI mutant and the R70W/L213LI double mutant, confirmed such dose-dependency (Figure 3E). Importantly, at a 1:1 DNA ratio (reminiscent of heterozygosity) there was still 22% residual NF-κB activation.

**Figure 4:**
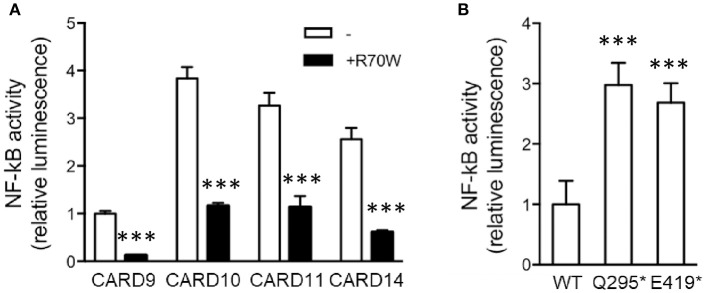
CARD9 premature stop mutants reveal the presence of an N-terminal auto-inhibition domain **(A)**. NF-κB luciferase reporter assay of R70W CARD9 co-transfected with auto-active mutants of CARD9, CARD10, CARD11, and CARD14, all lacking the C-terminal inhibitory domain. Luciferase values were expressed as fold induction compared to WT CARD9 alone. **(B)** NF-κB luciferase reporter assay of CARD9 constructs, harboring a premature stop after the first CC (Q295^*^) and the second CC (E419^*^) domain. Results shown in both panels are mean +/- standard deviation of 4 replicates. Statistical analysis was performed on reporter assay data with one-way ANOVA and Tukey's multiple comparison's post-testing. The most relevant statistical differences are shown: each CARD protein compared with and without CARD9 R70W in **(A)** and each mutant compared to WT CARD9 in **(B)**. A list of all *p*-values is provided in Supplemental Tables 5, 6. ^***^p < 0.001.

Considering the known structural homology and the potential for functional redundancy between CARD-CC proteins, we also assessed the effect of R70W CARD9 on auto-active mutants of CARD10, CARD11, and CARD14, all lacking the C-terminal inhibitory domain (Figure 4A). Surprisingly, upon co-transfection R70W CARD9 repressed NF-κB activity by all 4 auto-active CARD-CC proteins.

The proposed mechanism of the L225LI mutation in CARD11 is that it blocks intramolecular auto-inhibition from an inhibitory domain in the linker between the CC and the C-terminal domain (17). To investigate whether the sequence downstream of the CC domain(s) in CARD9 acts as an inhibitory domain in an analogous manner, we generated CARD9 constructs harboring a premature stop after the first CC (295^*^) and the second CC (419^*^). As expected, the two truncated forms show elevated activity compared to WT CARD9, indicating a common activation mechanism among CARD-CC proteins (Figure 4B). The Q295^*^ mutation and similar Q289^*^ are the two most common mutations occurring in patients with CARD9 deficiency (11 and 15 patients, respectively) (Supplemental Table 1). Auto-activation had already been shown for the Q295^*^ mutant (10) but we here propose the mechanism behind that observation.

### R70W CARD9 disrupts downstream NF-κB signaling by inhibiting BCL10 recruitment and filamentous network formation

**Figure 5:**
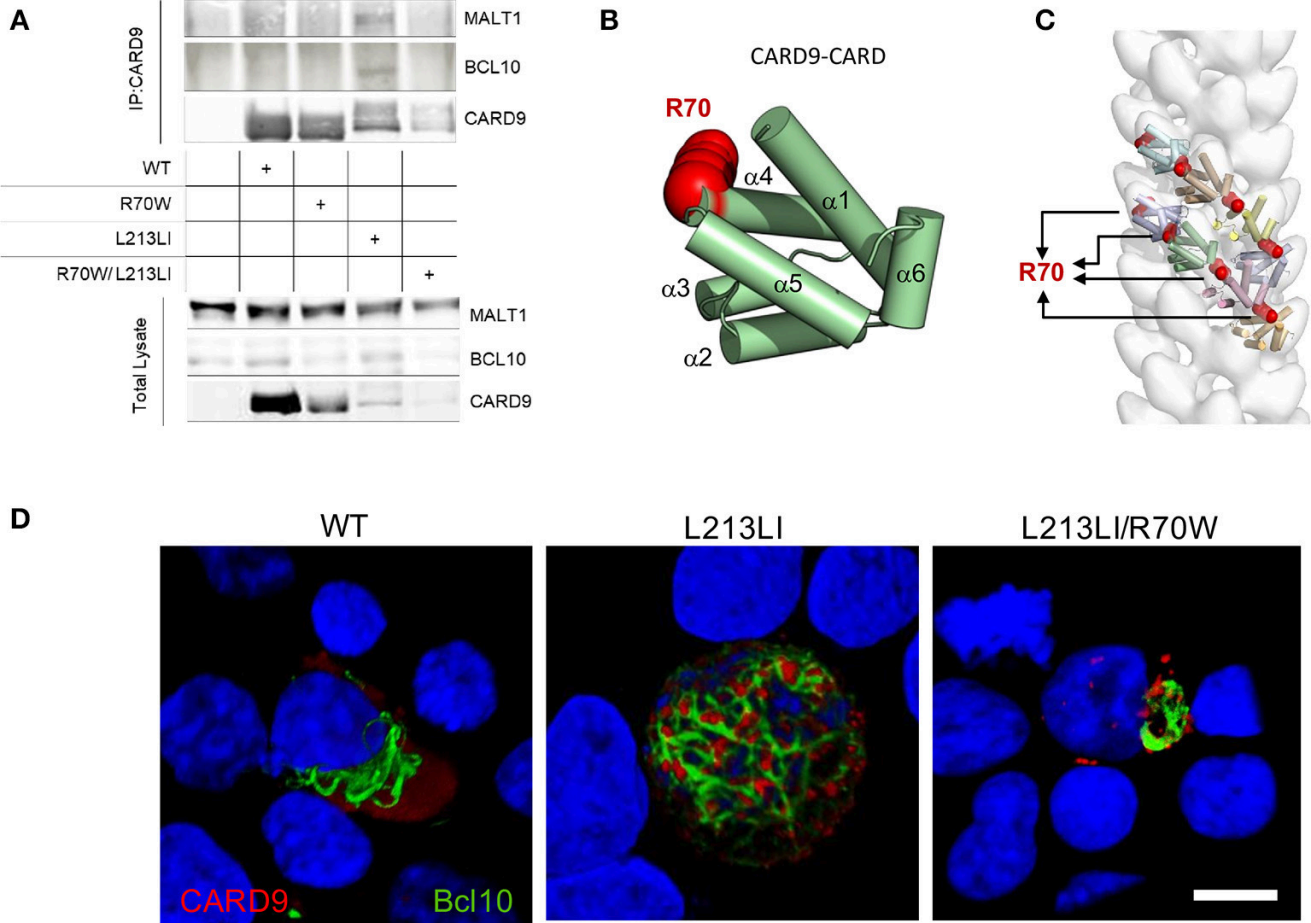
R70W CARD9 fails to pull down BCL10 and MALT1 and inhibits BCL10 filamentous network formation. **(A)** WT HEK293T cells were co-transfected with MALT1 and E-tagged BCL10 with indicated Flag-tagged CARD9 constructs. The CARD9 variants were immunoprecipitated with anti-Flag antibody and co-immunoprecipitation of BCL10 and MALT1 was detected (“IP”, upper panel) by anti-E-tag (BCL10) and anti-MALT1 antibody. CARD9 was detected with anti-Flag-HRP antibody. Input controls as immunoblotted on total lysate are shown in the lower panel. Density quantification of CARD9 on the total lysates is shown in Supplemental Figure 4. **(B)** A structural model for human CARD9 was created from structure-based sequence alignments of the sequence encoding the CARD domain of human CARD9 (uniprot Q9H257) and further improved computed via I-TASSER and QUARK. The R70 residue is depicted in red. **(C)** The homology model of the CARD domain of CARD9 was docked into a three-dimensional model representing the human BCL10 filament using the segment fitting algorithms implemented in Chimera. **(D)** E-tagged BCL10 was co-transfected with indicated Flag-tagged CARD9 constructs in un-stimulated HEK293T cells. Confocal imaging was performed with anti-E (BCL10, Green) and anti-Flag (CARD9, Red). 3D reconstructions of 50 Z-stacks are shown. Scale bar = 10μm.

To elucidate the underlying mechanism prohibiting R70W CARD9 from activating NF-κB, we studied its interactions with BCL10 and MALT1, which together with CARD9 form filamentous CBM complexes. As previously shown (28), without upstream activation, WT CARD9 failed to pull down BCL10 and MALT1, despite its activation of NF-κB in the reporter assays, presumably because that latter assay is more sensitive. To verify WT CARD9 could be activated by upstream stimuli, we used a setup similar to Figure 3A, but with upstream activation by PMA that activates protein kinase C upstream of CARD9. The results show that WT CARD9 is able to pull-down BCL10 and MALT1 upon activation (Supplemental Figure 3). Despite a decreased protein expression for the CARD9 mutants (Supplemental Figure 4), but in line with the reporter assay results, the GOF CARD9 mutant was exclusively able to pull-down BCL10 and MALT1. The R70W mutant did not pull down BCL10 and MALT1 and inhibited the GOF mutant to do so in the double mutant setup (Figure 5A), thus revealing that BCL10 recruitment is impaired by the R70W mutation. To rationalize the functional impact of the R70W mutation in CARD9 and to obtain complementary mechanistic insights, we leveraged structural information regarding the assembly principles of filamentous BCL10 and the CARD11-BCL10-MALT1 signalosome (15). N-terminal CARD domains sharing strong conservation in sequence and structure, constitute a hallmark of signalosome proteins, and are responsible for the ability of such proteins to oligomerize into intracellular filamentous structures (13–15). Given the paucity of structural information for the CARD domain of CARD9, we took advantage of the high sequence and structural homology shared by CARD domains to create a homology model for the CARD domain of CARD9 (Supplemental Figure 5 and main Figure 5B). In the first instance, this revealed that position R70 maps to the end of helix 4 (α4) at the surface of the helical CARD9 scaffold (Figure 5B). Therefore, we reasoned that radical mutation of R70 to a tryptophan (grantham score = 101), as is the case in the identified R70W CARD, could be deleterious for the ability of CARD9 to interact either with itself to form filaments, or to annex to BCL10 filaments to nucleate signaling-competent CBM signalosomes, or to interact with other partner proteins. Indeed, modeling of CARD9-CARD structures into the BCL10-CARD filament as revealed by electron microscopy (15), indicates that position R70 localizes at the heart of the interfaces of consecutive CARD9-CARD modules within the helical filament (Figure 5C). Thus, mutation of this position to any amino acid residue, and in particular to a radically different amino acid in terms of structural and chemical properties such as tryptophan, will be expected to abrogate the ability of CARD9-CARD to mediate filamentous forms of the protein.

Looking for evidence to substantiate our *in silico* predictions, we performed confocal imaging on HEK293T cells transfected with Flag-CARD9 and E-BCL10. Wild-type CARD9 was found as a diffuse signal neighboring BCL10 filaments, which were polarized at one side of the nucleus (Figure 5D left panel). Compared to WT, the GOF L213LI CARD9 mutant induced a clustering of CARD9 in dense spheroid structures ornamenting an intricate network of branched BCL10 filaments that stretched all over the cellular body (Figure 5D middle panel). In contrast, the GOF/LOF double mutant CARD9 failed to form a BCL10 network. Despite concentration of CARD9 in spheroid structures, suggesting that CARD9 itself was activated, the BCL10 filaments remained polarized at one side of the nucleus and failed to form a branched network all over the cell (Figure 5D right panel). This demonstrates that the R70W mutation indeed abrogates filamentous signalosome formation.

## Discussion

The adaptor protein CARD9 connects C-type lectin-receptor signals to the CBM signalosome to activate NF-κB upon fungal recognition by myeloid cells. Biallelic *CARD9* LOF mutations are associated with a wide range of fungal infections (Supplemental Table 1) and the here described R70W CARD9 patient cohort displays the same heterogeneity. This demonstrates that the nature of fungal infections a patient suffers from, is not determined by the type of *CARD9* mutation. The degree and severity of the presenting phenotype vary considerably between affected individuals and seem to aggravate with age with CMC always preceding invasive fungal disease (except in the case of F2 I:2 for whom this is unknown). Hence, an early diagnosis and accurate prophylaxis and follow-up are imperative to prevent CARD9 deficient patients from developing invasive infections.

The presence of a *CARD9* R70W founder mutation in the Turkish population from Afyonkarahisar/Eskişehir might simplify the laboratory diagnosis of CARD9 deficiency by targeted sequencing of the third exon. However, other mutations could be prevalent in that same population, since at least three patients of Turkish origin (though not precisely defined from which region) have been described with a Q295^*^ nonsense mutation (exon 6) and a clinical presentation similar to the patients described in this manuscript (29, 30). Targeted resequencing of the R70W mutation in 68 healthy Turkish individuals from the Istanbul region (results not shown) showed that the allele was absent there, suggesting that it may be geographically confined to the rural provinces of Afyonkarahisar and Eskişehir, where a tradition of consanguineous marriages promotes passing on of recessive disease (31). At this moment, we have no evidence that the R70W *CARD9* mutation would provide any beneficial traits to the population, and its presence could thus be a direct consequence of endogamy within a small and genetically homogenous population, where a detrimental recessive allele became common through the founder effect (32, 33). Earlier research has already shown a common founder origin for another *CARD9* mutation [Q289^*^; (7)] of middle-eastern origin, which raises the idea that CARD9 heterozygosity may offer a certain protective effect in populations from the greater Mediterranean basin. Larger genetic studies looking at carriership of rare *CARD9* variants in this and other populations may provide more substance to this hypothesis.

We confirmed earlier observations that R70W CARD9 fails to activate NF-κB (10). In addition, we uncovered that this is because the R70W mutation disrupts the interaction with the critical downstream signaling proteins, BCL10 and MALT1, thus preventing the formation of CBM signalosomes.

Remarkable was the inhibitory effect of the R70W allele seen *in vitro* upon co-transfection with WT and GOF CARD9, which stands in sharp contrast to the absence of symptoms in heterozygous R70W carriers. The lack of phenotype in heterozygous carriers can be explained by a dose-dependent activation of NF-κB. This is supported by previous observations (10) and our own that the R70W allele has a tendency to produce less protein compared to WT. From our dose-response curves, we know that the R70W mutation, when expressed in the double mutant with the GOF allele, at a 1:1 DNA ratio with the GOF single mutant, leaves about 22% residual NF-κB activity. Translated to heterozygotes, such residual NF-κB activity could explain the absence of disease. Based on the low expression levels of the R70W allele that we (Supplemental Figure 4) and others (10) observed, heterozygotes would have a relative expression of WT/mutant CARD9 somewhere in the range of 70/30 or higher, hence even more residual NF-kB activity could be suspected.

The key to the profound inhibiting effect of R70W CARD9 lies in the structural data, which revealed that position R70 in CARD9-CARD maps at the interface between successive CARD domains in CARD9 filaments. We have illustrated the dramatic impact of R70W CARD9 that is intrinsically unable to participate in CARD9 and BCL10 filaments to seed signalosome assembly in a cellular context. This is in line with previous observations that many of the BCL10-CARD and CARD11-CARD interfacial residues are crucial for BCL10 polymerization as well as CARD11-CBM complex formation-induced NF-κB activation (15). The same study further demonstrates how CARD11 nucleates BCL10 filaments in order to form an active CBM signalosome (15). They propose a scenario in which CARD11 forms short helical segments to nucleate BCL10 segments using its coiled-coil domain. Our high resolution confocal data corroborate that scenario of short spheroid-like segments. Most missense disease-causing mutations, like R70W, are situated in the CARD9 CARD- and CC-domains. Along the scenario proposed by Qiao et al., these mutations are highly likely to abolish BCL10 filamentous network formation. Such profound inhibition against WT proteins is a characteristic of mutants of highly oligomeric assemblies (15, 34), such as the inflammasome adaptor Apoptosis-associated speck-like protein (ASC), where ASC mutants with a non-functional CARD only assemble filaments but not specks, thus inhibiting formation of functional inflammasomes (35).

Our structural insights rationalize the use of the L213LI GOF mutant. Like many lymphoma-related mutations (17), it maps to the coiled-coil. Along the Qiao scenario, its gain-of-function action thus derives likely both from overcoming auto-inhibition and by enhancing oligomerization (18). The use of this GOF mutant as a model for the *in vitro* activation of CARD9 in HEK293T cells, allowed us to observe a much stronger inhibitory effect of the R70W mutant allele in comparison to an upstream activation model (10). The GOF CARD9 model system focuses on CARD9-CBM NF-κB activation, whereas upstream stimuli, be it Dectin1-ligation or PMA, would also activate endogenous other CARD-CC proteins (CARD10 in case of HEK293T cells) or alternative pathways, thereby possibly masking the full effect of the R70W allele.

The different CARD-CC proteins are to some extent functionally redundant: CARD10 was shown to be able to complement CARD11 deficiency in T cells (36), and there is evidence for CARD14-mediated zymosan signaling in keratinocytes (27). The R70W CARD9 allele also inhibited NF-κB activation by the three other CARD-CC proteins, CARD10, CARD11, and CARD14. This confirms the profound negative behavior of R70W CARD9 and demonstrates that hetero-multimerization can occur in the formation of a signaling-competent CBM signalosome. *In vivo*, however, CARD9 expression is confined to myeloid cells whereas CARD11 is expressed in lymphocytes and CARD10 and CARD14 mainly in different non-immune cells (www.biogps.org). These cell-type-specific expression profiles of the different CARD-CC proteins make it unlikely that a LOF CARD9 mutant would be able to influence the other CARD-CC signaling pathways *in vivo*. This is supported by the absence of symptoms others than fungal infections in CARD9 deficient patients.

The GOF CARD9 model system can be useful for future functional characterization of other disease-associated CARD9 mutations, as we illustrated for the Q295^*^ allele. The method is however not without limitations, since disease-causing mutations that influence the response of CARD9 to upstream activating signals would be expected to leave a GOF CARD9 mutant unaffected. In that case, co-expression of CARD9 mutants with upstream signaling components would still be the best option.

In conclusion, we here expand the R70W CARD9 cohort to 11 patients of five unrelated families from Turkish descent and provide evidence supporting a common founder origin of this mutation. We demonstrate that the R70W mutation disrupts the interaction with the critical downstream signaling partners BCL10 and MALT1, thus prohibiting the formation of CBM signalosomes and NF-κB signaling.

## Author contributions

MaD performed genetic analyses, analyzed genetic, and molecular analyses and wrote the manuscript. LH and DB assisted in genetic analyses. LV, EP, and MeD performed confocal analyses. ST assisted in molecular analyses. MM performed genetic analyses and provided patients samples. DK, BL, AA, LB, SvD, JY, and J-CG managed patients and provided clinical data. RB supervised molecular analyses. ED supervised genetic analyses. AP and J-LC provided patients samples and critically reviewed the data. SS performed structural analysis and critically reviewed the data. FH conceptualized the study, managed patients, provided clinical data and critically reviewed the data. JS conceptualized the study, supervised molecular analyses, performed data analysis and wrote the manuscript. MeD conceptualized the study, supervised experiments, performed data analysis and wrote the manuscript. All authors provided critical input and approved the final manuscript as submitted.

### Conflict of interest statement

The authors declare that the research was conducted in the absence of any commercial or financial relationships that could be construed as a potential conflict of interest.

## Abbreviations

AR: autosomal recessive
BCL10: B-cell CLL/lymphoma 10
CARD9: caspase recruitment domain family member 9
CBM: CARD-CC/BCL10/MALT1
CC: coiled-coil
CMC: chronic mucocutaneous candidiasis
Co-IP: co-immunoprecipitation
GOF: gain-of-function
IBD: Identity-by-descent
LOF: loss-of-function
LTT: lymphocyte transformation test
MALT1: mucosa-associated lymphoid tissue lymphoma translocation protein 1
NF-κB: nuclear factor kappa-light-chain-enhancer of activated B cells
PMA: phorbol 12-myristate 13-acetate
PWM: pokeweed mitogen
SNP: single-nucleotide polymorphism
WT: wild-type.
