# Supplementary material for: A CARD9 Founder Mutation Disrupts NF-κB Signaling by Inhibiting BCL10 and MALT1 Recruitment and Signalosome Formation
Source: Front Immunol. 2018 Oct 31;9:2366. doi: 10.3389/fimmu.2018.02366 (PMC6220056; doi:10.3389/fimmu.2018.02366)
Supplement: Supplementary file 1 [file Data_Sheet_1.docx]

**Supplemental information**

**A *CARD9* founder mutation disrupts NF-κB signaling by inhibiting BCL10 and MALT1 recruitment and signalosome formation**

Marieke De Bruyne, Msc ^1,2,3^, Levi Hoste, MD ^1,3^, Delfien J. Bogaert, MD, PhD ^1,2,3,4^, Lien Van den Bossche, PhD^1,5,6^, Simon J. Tavernier, MD, PhD^1, 4^, Eef Parthoens, MSc^6,7^, Mélanie Migaud, MSc ^8^, Deborah Konopnicki, MD, PhD ^9^, Jean Cyr Yombi, MD, PhD^10^, Bart N. Lambrecht, MD, PhD^4,11^, Sabine van Daele, MD, PhD ^3^, Ana Karina Alves de Medeiros, MD ^12^, Lieve Brochez, MD, PhD^12^, Rudi Beyaert, PhD ^13^, Elfride De Baere, MD, PhD ^2^, Anne Puel, PhD ^8^, Jean-Laurent Casanova, MD, PhD ^8,14^, Jean-Christophe Goffard, MD, PhD ^15^, Savvas N. Savvides, PhD ^5,6^, Filomeen Haerynck, MD, PhD ^1,3, $,*^, Jens Staal, PhD ^13, $^, Melissa Dullaers, PhD ^1,4, $^

^$^ These senior authors contributed equally.

***Corresponding author:** Filomeen Haerynck, Medical Research Building 2, Ghent University Hospital, Corneel Heymanslaan 10, B-9000 Ghent, Belgium; [filomeen.haerynck@uzgent.be](mailto:filomeen.haerynck@uzgent.be).

**SUPPLEMENTARY ONLINE MATERIAL**

**Case reports**

**Family 1** was previously described ^E^^1^^2^. Briefly, the index patient (F1 IV:5) is a 56-year-old male born to Turkish consanguineous parents from Eskişehir living in Belgium. The patient suffered from tinea capitis and tinea corporis on the trunk, arms and legs since the age of 8. At the time of first presentation, he also had oral candidiasis and onychomycosis. Skin cultures revealed different dermatophytes: *Trichophyton violaceum*, *T. verrucosum*, *T. rubrum* and *Malassezia furfur*. Fungal infections persisted despite treatment with various antifungal agents. At the age of 41, he developed a tumoral skin lesion on the chest. This lesion persisted for four years despite intensive antifungal treatment (itraconazole, ketoconazole and terbinafine) and needed to be surgically removed. Histopathological and immunohistochemical analysis revealed the presence of dermatophytes in the dermis and epidermis. Four years later, he presented dissemination of dermatophytes to the axillar lymph node, which was treated with antimycotics (terbinafine and griseofulvin). The proband’s grandmother (II:2) reportedly suffered from chronic dermatophytosis. The proband’s first son (V:2) suffered from CMC from the age of 8. The proband’s second son (V:3) presented with CMC at the age of 5, as well as chronic onychomycosis and hypoparathyroidism. Three months later, he developed right hemiparalysis due to *Candida* encephalitis and an internal carotid artery aneurysm with infarction. A computed tomography showed a hypodense zone in the left hemisphere. Analysis of cerebrospinal fluid showed 2900 leukocytes/mm³ (59% polymorphonuclear and 41% mononuclear cells) and glucose level of 68 mg/dL. *C. albicans* was repeatedly isolated from cerebrospinal fluid despite treatment with amphotericin B, flucytosine and fluconazole. Eventually he made a good recovery and the patient is no longer being followed by our clinic. Immunologic assessment showed normal lymphocyte counts (Table 1). Lymphocyte proliferation was weak upon *C. albicans* extract stimulation in all 3 patients. All 3 patients presented high serum IgE levels which was associated with eosinophilia in IV:5 and V:3. Further, IV:5 and V:3 both showed reduced IL-17 A and IL-22 production by CD4+ T helper cells. Additionally, IL-6 and granulocyte/macrophage colony-stimulating factor (GM-CSF) secretion by PBMC were reduced upon *C. albicans* and curdlan. Targeted next-generation sequencing of *CARD9* gene revealed bi-allelic R70W mutations.

The index patient (F2 V:2) of **family 2** is a 16-year-old girl born to Turkish consanguineous parents from Emirdağ (Afyonkarahisar) living in Belgium. Since the age of 7, she had recurrent oral candidiasis, onychomycosis and warts on the foot soles. Her 18-year-old brother (III:1) suffered from tinea corporis and tinea pedis on the abdomen and groin. Skin cultures revealed *Trichophyton rubrum*. The 10-year-old brother (III:3) presented with recurrent oral candidiasis since the age of 2,5 years. He also developed onychomycosis and recurrent limited warts on the foot soles. All patients responded well to standard antifungal treatment (miconazole, itraconazole, fluconazole and terbinafine) and they were given a maintenance therapy with fluconazole to prevent invasive fungal infections. Although the two younger sibs (III:2, III:3) refused further fluconazole prophylaxis in the past year, they are doing well without outbreaks of fungal infections. At the age of 48, the paternal grandmother (I:2) was diagnosed with hepatic blastomycosis after a splenectomy for unknown etiology. The infection persisted despite treatment with itraconazole for two years. Therefore and because of serious side effects of amphotericin B treatment, the patient was hospitalized twice for further follow-up and treatment. At that time, a puncture of the retro-peritoneal glands revealed an *Aspergillus fumigatus* infection, which eventually lead to fibrosis and ureter stenosis. Treatment with echinocandin and intravenous itraconazole lead to resolution of the infection. Additionally, she suffered from transient hypothyroidism, diabetes and osteoporosis. The patient has been clinical stable for 4 years now, and she is no longer in clinical follow-up. The maternal grandmother (I:4) reportedly has thyroid problems without CMC. Unfortunately, she is living in Turkey so that further investigation was not possible. Other family members are currently healthy. Immunologic investigation showed normal lymphocyte counts (Table 1). Lymphocyte proliferation was weak upon *C. Albicans* extract stimulation in patients III:2 and III:3, which also presented reduced IL-17 A and IL-22 production by CD4+ T helper cells. In addition, IL-6 secretion by PBMC was reduced upon *C. Albicans*. Identity-by-descent (IBD) mapping was carried out in two affected individuals (III:2, III:3) and revealed 10 regions (>1Mb) to be homozygous in both affected individuals. The second-largest IBD region (3,3 Mb) contained *CARD9*. Sanger sequencing of *CARD9* identified homozygosity for R70W. Segregation analysis by Sanger sequencing of *CARD9* exon 3 in the available family members confirmed segregation with disease in the family.

**Family 3** was previously reported ^E10^. A 42-year-old woman (F3 II.2) living in France and born to consanguineous Turkish parents originating from Afyonkarahisar, presented at 39 years of age with *Candida* *albicans*–induced meningitis and brain abscesses. She had recurrent vulvovaginal candidiasis, with episodes occurring about 5 times per year since the age of 36 years. She presented with headache, persistent fever, and vomiting. She then displayed an altered mental state, right-arm paresis, and facial palsy. Brain magnetic resonance imaging (MRI) provided evidence of an infiltrative frontal lesion with a mass effect and contrast enhancement with ventricular dilation. Lumbar puncture revealed the presence of 1100 leukocytes/mm3 (80% lymphocytes and 16% eosinophils) in the cerebrospinal fluid (CSF), together with increased protein levels up to 1.53 g/L and hypoglycorrhachia of 1.5 mmol/L. CSF pressure was high at up to 25 cm H2O. Three lumbar punctures were performed, and *C. albicans* grew from the CSF samples collected. Brain biopsy showed yeasts and numerous pseudohyphae in giant cell granulomas with necrosis. A culture of the biopsy sample was positive for *C. albicans*. Abdominal and thoracic computed tomography (CT) and transesophageal echocardiography provided no evidence for the dissemination of *C. albicans* infection. Immunologic explorations showed normal CD4+ T, CD8+ T, and natural killer (NK) lymphocyte counts and B-cell lymphopenia at 4% (56 cells/mL). T-lymphocyte proliferations were normal in response to PHA and antigens (tuberculin and candidin). Leukocyte oxidative burst, as assessed by dihydrorhodamine (DHR) tests, was normal, and IgG, IgA, and IgM levels were also normal. The infection was cured by 2 months of combined intravenous antifungal therapy combining liposomal amphotericin B and 5-fluorocytosine, which was subsequently replaced with oral fluconazole. A cerebral shunt was performed to treat intracranial hypertension. Two years later, fluconazole treatment is continuing, and the patient is alive without sequel. Neither her parents nor her siblings and children have had any severe infectious disease. Targeted next-generation sequencing of *CARD9* gene revealed bi-allelic missense mutations R70W.

**Family 4** was previously reported ^E10^. The index patient (F4 II.1) is an 8-year-old girl born to a Turkish family living in Belgium. She had chronic thrush and onychomycosis since the age of 5 years. At the age of 7 years, she presented fever for several weeks, with headache and vomiting. CSF analysis provided evidence of *C. albicans*-induced meningitis but with 920 cells/mm3, 20% of which were eosinophils. Brain MRI revealed the presence of 2 lesions of 11 and 6 mm in diameter, respectively. Medullary MRI revealed several enhancing lesions. *C. albicans* grew from nail and buccal samples. The patient was treated with liposomal amphotericin B for 2 weeks and then fluconazole, with a positive outcome. Soon after fluconazole treatment, a relapse occurred, with fever, headache, and vomiting. CSF culture was sterile but with 1520 cells/mm3, mostly eosinophils (60%). Symptoms improved with liposomal amphotericin B treatment, which was replaced after 5 months with fluconazole because of renal failure related to liposomal amphotericin B. Lesions were controlled 6 months after starting fluconazole. Brain lesions were in regression, whereas medullar lesions remained unchanged. The patient’s parents did not have any severe infectious disease. Targeted next-generation sequencing of *CARD9* gene revealed bi-allelic missense mutations R70W.

The index patient of **family 5** (F5 II:2) is a 35 year-old women presenting in November 2015 with a 13 cm mesenteric mass while 29 weeks pregnant with flank pain and significant weight loss since 2 months. On physical examination, the patient was afebrile but very sick with severe pain, grey skin and oral thrush. Her laboratory test showed mild inflammation and cholestasis (CRP 58 mg/L, alkaline phosphate 325 UI/L). She had had previous oral thrushes, vaginal and skin infections responding well to itraconazole therapy. She was born in Belgium from non-consanguineous Turkish parents originating from Emirdağ. One of her sister had died recently from gastric cancer at 28 years-old and another sister was taking fluconazole for life because of recurrent *Candida* bone infections followed in another hospital.

The mesenteric mass, probably several agglutinated lymphadenitis with calcification, could not be removed during surgery because of inclusion of mesenteric vessels but the biopsy showed granuloma, eosinophils infiltrates and fungal hyphae compatible with aspergillosis infection. A treatment with intravenous voriconazole was started for 14 days. All biopsy cultures for aerobic and anaerobic bacteria, mycobacteria, mold and yeasts and PCR for tuberculosis and *Aspergillus* were negative; however, biopsy PCR was positive for *Candida albicans*. The patient’s condition and laboratory abnormalities dramatically improved rapidly after starting voriconazole with appetite recovery, weight increase and pain decrease. Voriconazole was continued orally for a total duration of 6 months up to July 2016. She gave birth to a healthy girl in March 2016. The repeated magnetic resonances showed first a decrease in the mass size from 13 to 11 cm, then stabilization to 11 cm diameter. A Pet-scan performed after delivery showed mild heterogeneous hypermetabolism that had disappeared 3 months later. At the last visit in September 2017, the patient had maintained her normal weight, still had a pulsatile and palpable umbilical mass with moderate flank pain requiring daily paracetamol or oxycodone; she had also recurrent skin mycosis although she receives fluconazole 200 mg per day as prophylaxis since July 2016. A magnetic resonance performed on April 2017 showed a stable lesion of 11 cm.

Patient F5 II.6 is a 43-year-old women with a long history of invasive *Candida albicans* infection that started at the age of 8 with a tibial osteomyelitis and a right kidney abscess requiring nephrectomy. The patient remained in remission after a long treatment course with fluconazole until the age of 18 when she presented with bilateral hip arthritis for which we are lacking information. In 2003, she came back to the hospital with worsening right knee pain. Synovectomy, debridement and washouts were performed. Culture disclosed a new infection with *Candida albicans* remaining susceptible to Fluconazole. Fluconazole was prescribed for a lifelong therapy. Bilateral arthroplasty of hips and of the right knee was performed in 2006, 2009 and 2016 without complications.

**Supplemental Table 1: Overview of published *CARD9* patients**

**Supplemental Table 2: Statistical analysis on NF-κB transcriptional activity results for figure 3B**

|  | **Mean 1** | **Mean 2** | **Adjusted p-value** |
| --- | --- | --- | --- |
| WT vs. WT+MALT1 | 1,037 | 2,39 | <0.0001 |
| WT vs. R70W | 1,037 | 0,7471 | 0,0475 |
| WT vs. R70W+MALT1 | 1,037 | 0,5054 | 0,0002 |
| WT vs. WT/R70W | 1,037 | 1,077 | 0,9975 |
| WT vs. WT/R70W +MALT1 | 1,037 | 0,7702 | 0,0779 |
| WT+MALT1 vs. R70W | 2,39 | 0,7471 | <0.0001 |
| WT+MALT1 vs. R70W+MALT1 | 2,39 | 0,5054 | <0.0001 |
| WT+MALT1 vs. WT/R70W | 2,39 | 1,077 | <0.0001 |
| WT+MALT1 vs. WT/R70W +MALT1 | 2,39 | 0,7702 | <0.0001 |
| R70W vs. R70W+MALT1 | 0,7471 | 0,5054 | 0,1302 |
| R70W vs. WT/R70W | 0,7471 | 1,077 | 0,0194 |
| R70W vs. WT/R70W +MALT1 | 0,7471 | 0,7702 | 0,9998 |
| R70W+MALT1 vs. WT/R70W | 0,5054 | 1,077 | <0.0001 |
| R70W+MALT1 vs. WT/R70W +MALT1 | 0,5054 | 0,7702 | 0,0814 |
| WT/R70W vs. WT/R70W +MALT1 | 1,077 | 0,7702 | 0,0327 |

**Supplemental Table 3: Statistical analysis on NF-κB transcriptional activity results for figure 3D**

|  | **Mean 1** | **Mean 2** | **adjusted p-value** |
| --- | --- | --- | --- |
| WT vs. WT+MALT1 | 1 | 3,85 | 0,6706 |
| WT vs. R70W | 1 | 0,275 | >0.9999 |
| WT vs. R70W +MALT1 | 1 | 0,3 | >0.9999 |
| WT vs. L213LI | 1 | 4,575 | 0,3448 |
| WT vs. L213LI + MALT1 | 1 | 52,23 | <0.0001 |
| WT vs. L213LI/R70W | 1 | 0,25 | >0.9999 |
| WT vs. L213LI/R70W +MALT1 | 1 | 0,45 | >0.9999 |
| WT vs. L213LI +R70W | 1 | 0,5 | >0.9999 |
| WT vs. L213LI +R70W +MALT1 | 1 | 0,275 | >0.9999 |
| WT vs. L213LI+R70W/L213LI | 1 | 1,525 | >0.9999 |
| WT vs. L213LI+R70W/L213LI +MALT1 | 1 | 4,45 | 0,3958 |
| WT+MALT1 vs. R70W | 3,85 | 0,275 | 0,3448 |
| WT+MALT1 vs. R70W +MALT1 | 3,85 | 0,3 | 0,3547 |
| WT+MALT1 vs. L213LI | 3,85 | 4,575 | >0.9999 |
| WT+MALT1 vs. L213LI + MALT1 | 3,85 | 52,23 | <0.0001 |
| WT+MALT1 vs. L213LI/R70W | 3,85 | 0,25 | 0,335 |
| WT+MALT1 vs. L213LI/R70W +MALT1 | 3,85 | 0,45 | 0,4172 |
| WT+MALT1 vs. L213LI +R70W | 3,85 | 0,5 | 0,4391 |
| WT+MALT1 vs. L213LI +R70W +MALT1 | 3,85 | 0,275 | 0,3448 |
| WT+MALT1 vs. L213LI+R70W/L213LI | 3,85 | 1,525 | 0,8754 |
| WT+MALT1 vs. L213LI+R70W/L213LI +MALT1 | 3,85 | 4,45 | >0.9999 |
| R70W vs. R70W +MALT1 | 0,275 | 0,3 | >0.9999 |
| R70W vs. L213LI | 0,275 | 4,575 | 0,1313 |
| R70W vs. L213LI + MALT1 | 0,275 | 52,23 | <0.0001 |
| R70W vs. L213LI/R70W | 0,275 | 0,25 | >0.9999 |
| R70W vs. L213LI/R70W +MALT1 | 0,275 | 0,45 | >0.9999 |
| R70W vs. L213LI +R70W | 0,275 | 0,5 | >0.9999 |
| R70W vs. L213LI +R70W +MALT1 | 0,275 | 0,275 | >0.9999 |
| R70W vs. L213LI+R70W/L213LI | 0,275 | 1,525 | 0,9988 |
| R70W vs. L213LI+R70W/L213LI +MALT1 | 0,275 | 4,45 | 0,1579 |
| R70W +MALT1 vs. L213LI | 0,3 | 4,575 | 0,1363 |
| R70W +MALT1 vs. L213LI + MALT1 | 0,3 | 52,23 | <0.0001 |
| R70W +MALT1 vs. L213LI/R70W | 0,3 | 0,25 | >0.9999 |
| R70W +MALT1 vs. L213LI/R70W +MALT1 | 0,3 | 0,45 | >0.9999 |
| R70W +MALT1 vs. L213LI +R70W | 0,3 | 0,5 | >0.9999 |
| R70W +MALT1 vs. L213LI +R70W +MALT1 | 0,3 | 0,275 | >0.9999 |
| R70W +MALT1 vs. L213LI+R70W/L213LI | 0,3 | 1,525 | 0,999 |
| R70W +MALT1 vs. L213LI+R70W/L213LI +MALT1 | 0,3 | 4,45 | 0,1637 |
| L213LI vs. L213LI + MALT1 | 4,575 | 52,23 | <0.0001 |
| L213LI vs. L213LI/R70W | 4,575 | 0,25 | 0,1265 |
| L213LI vs. L213LI/R70W +MALT1 | 4,575 | 0,45 | 0,1697 |
| L213LI vs. L213LI +R70W | 4,575 | 0,5 | 0,1821 |
| L213LI vs. L213LI +R70W +MALT1 | 4,575 | 0,275 | 0,1313 |
| L213LI vs. L213LI+R70W/L213LI | 4,575 | 1,525 | 0,5774 |
| L213LI vs. L213LI+R70W/L213LI +MALT1 | 4,575 | 4,45 | >0.9999 |
| L213LI + MALT1 vs. L213LI/R70W | 52,23 | 0,25 | <0.0001 |
| L213LI + MALT1 vs. L213LI/R70W +MALT1 | 52,23 | 0,45 | <0.0001 |
| L213LI + MALT1 vs. L213LI +R70W | 52,23 | 0,5 | <0.0001 |
| L213LI + MALT1 vs. L213LI +R70W +MALT1 | 52,23 | 0,275 | <0.0001 |
| L213LI + MALT1 vs. L213LI+R70W/L213LI | 52,23 | 1,525 | <0.0001 |
| L213LI + MALT1 vs. L213LI+R70W/L213LI +MALT1 | 52,23 | 4,45 | <0.0001 |
| L213LI/R70W vs. L213LI/R70W +MALT1 | 0,25 | 0,45 | >0.9999 |
| L213LI/R70W vs. L213LI +R70W | 0,25 | 0,5 | >0.9999 |
| L213LI/R70W vs. L213LI +R70W +MALT1 | 0,25 | 0,275 | >0.9999 |
| L213LI/R70W vs. L213LI+R70W/L213LI | 0,25 | 1,525 | 0,9986 |
| L213LI/R70W vs. L213LI+R70W/L213LI +MALT1 | 0,25 | 4,45 | 0,1523 |
| L213LI/R70W +MALT1 vs. L213LI +R70W | 0,45 | 0,5 | >0.9999 |
| L213LI/R70W +MALT1 vs. L213LI +R70W +MALT1 | 0,45 | 0,275 | >0.9999 |
| L213LI/R70W +MALT1 vs. L213LI+R70W/L213LI | 0,45 | 1,525 | 0,9997 |
| L213LI/R70W +MALT1 vs. L213LI+R70W/L213LI +MALT1 | 0,45 | 4,45 | 0,2021 |
| L213LI +R70W vs. L213LI +R70W +MALT1 | 0,5 | 0,275 | >0.9999 |
| L213LI +R70W vs. L213LI+R70W/L213LI | 0,5 | 1,525 | 0,9998 |
| L213LI +R70W vs. L213LI+R70W/L213LI +MALT1 | 0,5 | 4,45 | 0,2162 |
| L213LI +R70W +MALT1 vs. L213LI+R70W/L213LI | 0,275 | 1,525 | 0,9988 |
| L213LI +R70W +MALT1 vs. L213LI+R70W/L213LI +MALT1 | 0,275 | 4,45 | 0,1579 |
| L213LI+R70W/L213LI vs. L213LI+R70W/L213LI +MALT1 | 1,525 | 4,45 | 0,636 |

**Supplemental Table 4: Statistical analysis on NF-κB transcriptional activity results for figure 3E**

|  | **Mean 1** | **Mean 2** | **adjusted p-value** |
| --- | --- | --- | --- |
| 0 vs. 5 | 1 | 0,8229 | 0,5994 |
| 0 vs. 12.5 | 1 | 0,7695 | 0,2697 |
| 0 vs. 25 | 1 | 0,5317 | 0,0011 |
| 0 vs. 37.5 | 1 | 0,3827 | <0.0001 |
| 0 vs. 50 | 1 | 0,2241 | <0.0001 |
| 0 vs. 62.5 | 1 | 0,08689 | <0.0001 |
| 0 vs. 75 | 1 | 0,01461 | <0.0001 |
| 0 vs. 87.5 | 1 | 0,002376 | <0.0001 |
| 0 vs. 100 | 1 | 0,002489 | <0.0001 |
| 5 vs. 12.5 | 0,8229 | 0,7695 | 0,9997 |
| 5 vs. 25 | 0,8229 | 0,5317 | 0,079 |
| 5 vs. 37.5 | 0,8229 | 0,3827 | 0,0022 |
| 5 vs. 50 | 0,8229 | 0,2241 | <0.0001 |
| 5 vs. 62.5 | 0,8229 | 0,08689 | <0.0001 |
| 5 vs. 75 | 0,8229 | 0,01461 | <0.0001 |
| 5 vs. 87.5 | 0,8229 | 0,002376 | <0.0001 |
| 5 vs. 100 | 0,8229 | 0,002489 | <0.0001 |
| 12.5 vs. 25 | 0,7695 | 0,5317 | 0,2364 |
| 12.5 vs. 37.5 | 0,7695 | 0,3827 | 0,0081 |
| 12.5 vs. 50 | 0,7695 | 0,2241 | 0,0002 |
| 12.5 vs. 62.5 | 0,7695 | 0,08689 | <0.0001 |
| 12.5 vs. 75 | 0,7695 | 0,01461 | <0.0001 |
| 12.5 vs. 87.5 | 0,7695 | 0,002376 | <0.0001 |
| 12.5 vs. 100 | 0,7695 | 0,002489 | <0.0001 |
| 25 vs. 37.5 | 0,5317 | 0,3827 | 0,7862 |
| 25 vs. 50 | 0,5317 | 0,2241 | 0,0544 |
| 25 vs. 62.5 | 0,5317 | 0,08689 | 0,0019 |
| 25 vs. 75 | 0,5317 | 0,01461 | 0,0003 |
| 25 vs. 87.5 | 0,5317 | 0,002376 | 0,0002 |
| 25 vs. 100 | 0,5317 | 0,002489 | 0,0002 |
| 37.5 vs. 50 | 0,3827 | 0,2241 | 0,7255 |
| 37.5 vs. 62.5 | 0,3827 | 0,08689 | 0,0711 |
| 37.5 vs. 75 | 0,3827 | 0,01461 | 0,0129 |
| 37.5 vs. 87.5 | 0,3827 | 0,002376 | 0,0095 |
| 37.5 vs. 100 | 0,3827 | 0,002489 | 0,0095 |
| 50 vs. 62.5 | 0,2241 | 0,08689 | 0,8515 |
| 50 vs. 75 | 0,2241 | 0,01461 | 0,3843 |
| 50 vs. 87.5 | 0,2241 | 0,002376 | 0,3145 |
| 50 vs. 100 | 0,2241 | 0,002489 | 0,3151 |
| 62.5 vs. 75 | 0,08689 | 0,01461 | 0,9972 |
| 62.5 vs. 87.5 | 0,08689 | 0,002376 | 0,9915 |
| 62.5 vs. 100 | 0,08689 | 0,002489 | 0,9916 |
| 75 vs. 87.5 | 0,01461 | 0,002376 | >0.9999 |
| 75 vs. 100 | 0,01461 | 0,002489 | >0.9999 |
| 87.5 vs. 100 | 0,002376 | 0,002489 | >0.9999 |

**Supplemental Table 5: Statistical analysis on NF-κB transcriptional activity results for figure 4A**

|  | **Mean 1** | **Mean 2** | **adjusted p-value** |
| --- | --- | --- | --- |
| CARD9 - vs. CARD9+R70W | 1 | 0,1233 | 0,0003 |
| CARD9 - vs. CARD10 - | 1 | 3,837 | <0.0001 |
| CARD9 - vs. CARD10 +R70W | 1 | 1,16 | 0,9446 |
| CARD9 - vs. CARD11 - | 1 | 3,267 | <0.0001 |
| CARD9 - vs. CARD11 + R70W | 1 | 1,133 | 0,9785 |
| CARD9 - vs. CARD14 - | 1 | 2,56 | <0.0001 |
| CARD9 - vs. CARD14 + R70W | 1 | 0,6067 | 0,181 |
| CARD9+R70W vs. CARD10 - | 0,1233 | 3,837 | <0.0001 |
| CARD9+R70W vs. CARD10 +R70W | 0,1233 | 1,16 | <0.0001 |
| CARD9+R70W vs. CARD11 - | 0,1233 | 3,267 | <0.0001 |
| CARD9+R70W vs. CARD11 + R70W | 0,1233 | 1,133 | <0.0001 |
| CARD9+R70W vs. CARD14 - | 0,1233 | 2,56 | <0.0001 |
| CARD9+R70W vs. CARD14 + R70W | 0,1233 | 0,6067 | 0,0603 |
| CARD10 - vs. CARD10 +R70W | 3,837 | 1,16 | <0.0001 |
| CARD10 - vs. CARD11 - | 3,837 | 3,267 | 0,0192 |
| CARD10 - vs. CARD11 + R70W | 3,837 | 1,133 | <0.0001 |
| CARD10 - vs. CARD14 - | 3,837 | 2,56 | <0.0001 |
| CARD10 - vs. CARD14 + R70W | 3,837 | 0,6067 | <0.0001 |
| CARD10 +R70W vs. CARD11 - | 1,16 | 3,267 | <0.0001 |
| CARD10 +R70W vs. CARD11 + R70W | 1,16 | 1,133 | >0.9999 |
| CARD10 +R70W vs. CARD14 - | 1,16 | 2,56 | <0.0001 |
| CARD10 +R70W vs. CARD14 + R70W | 1,16 | 0,6067 | 0,024 |
| CARD11 - vs. CARD11 + R70W | 3,267 | 1,133 | <0.0001 |
| CARD11 - vs. CARD14 - | 3,267 | 2,56 | 0,003 |
| CARD11 - vs. CARD14 + R70W | 3,267 | 0,6067 | <0.0001 |
| CARD11 + R70W vs. CARD14 - | 1,133 | 2,56 | <0.0001 |
| CARD11 + R70W vs. CARD14 + R70W | 1,133 | 0,6067 | 0,0342 |
| CARD14 - vs. CARD14 + R70W | 2,56 | 0,6067 | <0.0001 |

**Supplemental Table 6: Statistical analysis on NF-κB transcriptional activity results for figure 4B**

|  | **Mean 1** | **Mean 2** | **adjusted p-value** |
| --- | --- | --- | --- |
| WT vs. Q295* | 1 | 2,978 | <0.0001 |
| WT vs. E419* | 1 | 2,682 | 0,0003 |
| Q295* vs. E419* | 2,978 | 2,682 | 0,5036 |

**Supplemental Table 1: Overview of published CARD9 patients**

* if different from origin: ** patients not genotyped (-) because no DNA available; *** known (+) or if empty, no, familial history and/or consanguinity; **** references of published cases (see supplemental references); M: male; F: female; CARD: caspase recruitment domain; CC: coiled-coil domain; NA: not available; bold: patients included in the current study.

**Supplemental Tables 2-6: Statistical analysis on NF-κB transcriptional activity results for figures 3B, 3D, 3E, 4A and 4B**

Mean 1 and Mean 2 represent the relative luminescence values. Luciferase values were normalized against β-galactosidase and expressed as fold induction compared to WT CARD9 without MALT1. Statistical analysis was performed on the reporter assay data with one-way ANOVA and Tukey’s multiple comparison’s post-testing (adjusted p-value)

**SUPPLEMENTAL** **REFERENCES**

E1. Lanternier F, Pathan S, Vincent QB, et al. Deep dermatophytosis and inherited CARD9 deficiency. *N Engl J Med*. 2013;369(18):1704-1714. doi:10.1056/NEJMoa1208487.

E2. Lanternier F, Barbati E, Meinzer U, et al. Inherited CARD9 deficiency in 2 unrelated patients with invasive Exophiala infection. *J Infect Dis*. 2015;211(8):1241-1250. doi:10.1093/infdis/jiu412.

E3. Drewniak A, Gazendam RP, Tool ATJ, et al. Invasive fungal infection and impaired neutrophil killing in human CARD9 deficiency. *Blood*. 2013;121(13):2385-2392. doi:10.1182/blood-2012-08-450551.

E4. Yan XX, Yu CP, Fu XA, et al. CARD9 mutation linked to Corynespora cassiicola infection in a Chinese patient. *Br J Dermatol*. 2016;174(1):176-179. doi:10.1111/bjd.14082.

E5. Jachiet M, Lanternier F, Rybojad M, et al. Posaconazole treatment of extensive skin and nail dermatophytosis due to autosomal recessive deficiency of CARD9. *JAMA dermatology*. 2015;151(2):192-194. doi:10.1001/jamadermatol.2014.2154.

E6. Drummond RA, Collar AL, Swamydas M, et al. CARD9-Dependent Neutrophil Recruitment Protects against Fungal Invasion of the Central Nervous System. *PLoS Pathog*. 2015;11(12):e1005293. doi:10.1371/journal.ppat.1005293.

E7. Gavino C, Cotter A, Lichtenstein D, et al. CARD9 deficiency and spontaneous central nervous system candidiasis: Complete clinical remission with GM-CSF therapy. *Clin Infect Dis*. 2014;59(1):81-84. doi:10.1093/cid/ciu215.

E8. Gavino C, Hamel N, Zeng J Bin, et al. Impaired RASGRF1/ERK-mediated GM-CSF response characterizes CARD9 deficiency in French-Canadians. *J Allergy Clin Immunol*. 2016;137(4):1178-1188.e7. doi:10.1016/j.jaci.2015.09.016.

E9. Glocker E-O, Hennigs A, Nabavi M, et al. A homozygous CARD9 mutation in a family with susceptibility to fungal infections. *N Engl J Med*. 2009;361(18):1727-1735. doi:10.1056/NEJMoa0810719.

E10. Lanternier F, Mahdaviani SA, Barbati E, et al. Inherited CARD9 deficiency in otherwise healthy children and adults with Candida species-induced meningoencephalitis, colitis, or both. *J Allergy Clin Immunol*. 2015;135(6):1558-68.e2. doi:10.1016/j.jaci.2014.12.1930.

E11. Grumach AS, de Queiroz-Telles F, Migaud M, et al. A homozygous CARD9 mutation in a Brazilian patient with deep dermatophytosis. *J Clin Immunol*. 2015;35(5):486-490. doi:10.1007/s10875-015-0170-4.

E12. Alves de Medeiros AK, Lodewick E, Bogaert DJA, et al. Chronic and Invasive Fungal Infections in a Family with CARD9 Deficiency. *J Clin Immunol*. 2016:1-6. doi:10.1007/s10875-016-0255-8.

E13. Celmeli F, Oztoprak N, Turkkahraman D, et al. Successful Granulocyte Colony-stimulating Factor Treatment of Relapsing Candida albicans Meningoencephalitis Caused by CARD9 Deficiency. *Pediatr Infect Dis J*. 2016;35(4):428-431. doi:10.1097/INF.0000000000001028.

E14. Herbst M, Gazendam R, Reimnitz D, et al. Chronic Candida albicans Meningitis in a 4-Year-Old Girl with a Homozygous Mutation in the CARD9 Gene (Q295X). *Pediatr Infect Dis J*. 2015;34(9):999-1002. doi:10.1097/INF.0000000000000736.

E15. Wang X, Wang W, Lin Z, et al. CARD9 mutations linked to subcutaneous phaeohyphomycosis and TH17 cell deficiencies. *J Allergy Clin Immunol*. 2014;133(3):905-8.e3. doi:10.1016/j.jaci.2013.09.033.

E16. Jones N, Garcez T, Newman W, Denning D. Endogenous *Candida* endophthalmitis and osteomyelitis associated with CARD9 deficiency. *BMJ Case Rep*. 2016;(May):bcr2015214117. doi:10.1136/bcr-2015-214117.

E17. Rieber N, Gazendam RP, Freeman AF, et al. Extrapulmonary Aspergillus infection in patients with CARD9 deficiency. *JCI insight*. 2016;1(17):e89890. doi:10.1172/jci.insight.89890.

E18. Boudghene Stambouli O, Amrani N, Boudghéne Stambouli K, Bouali F. Dermatophytic disease with deficit in CARD9: A new case with a brain impairment. *J Mycol Med*. 2017;27(2):250-253. doi:10.1016/j.mycmed.2017.01.001.

E19. Gavino C, Mellinghoff S, Cornely OA, et al. Novel Bi-allelic Splice Mutations in CARD9 Causing Adult-onset Candida Endophthalmitis. *Mycoses*. 2017;12(10):3218-3221. doi:10.1111/myc.12701.
