# Supplementary figures and images for: A CARD9 Founder Mutation Disrupts NF-κB Signaling by Inhibiting BCL10 and MALT1 Recruitment and Signalosome Formation
Source: Front Immunol. 2018 Oct 31;9:2366. doi: 10.3389/fimmu.2018.02366 (PMC6220056; doi:10.3389/fimmu.2018.02366)

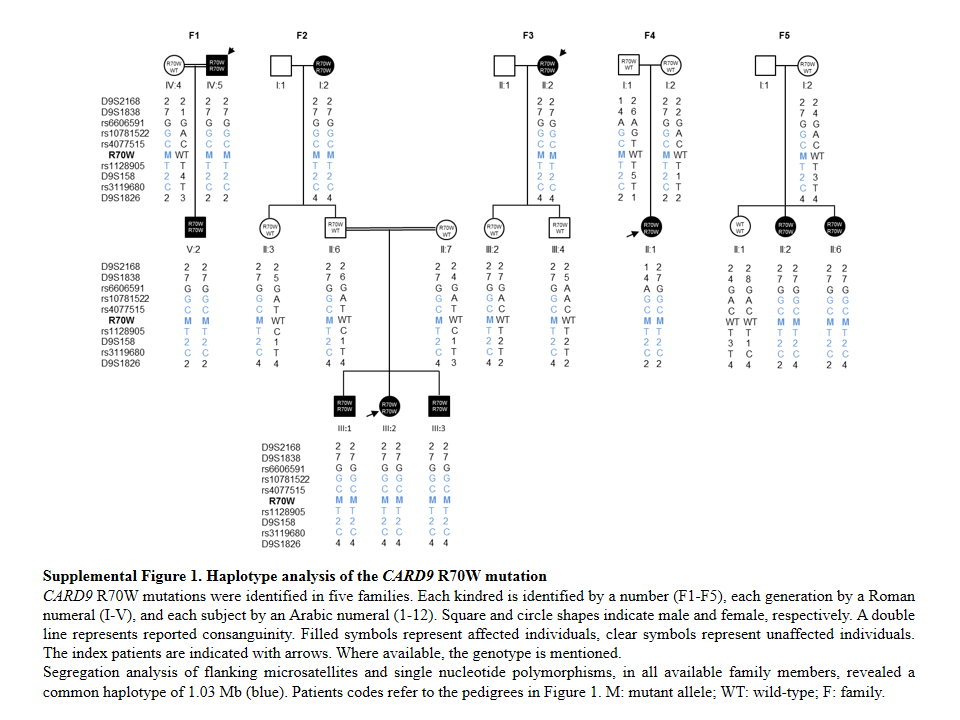

Supplement: Supplementary file 2 [file Image_1.TIF]

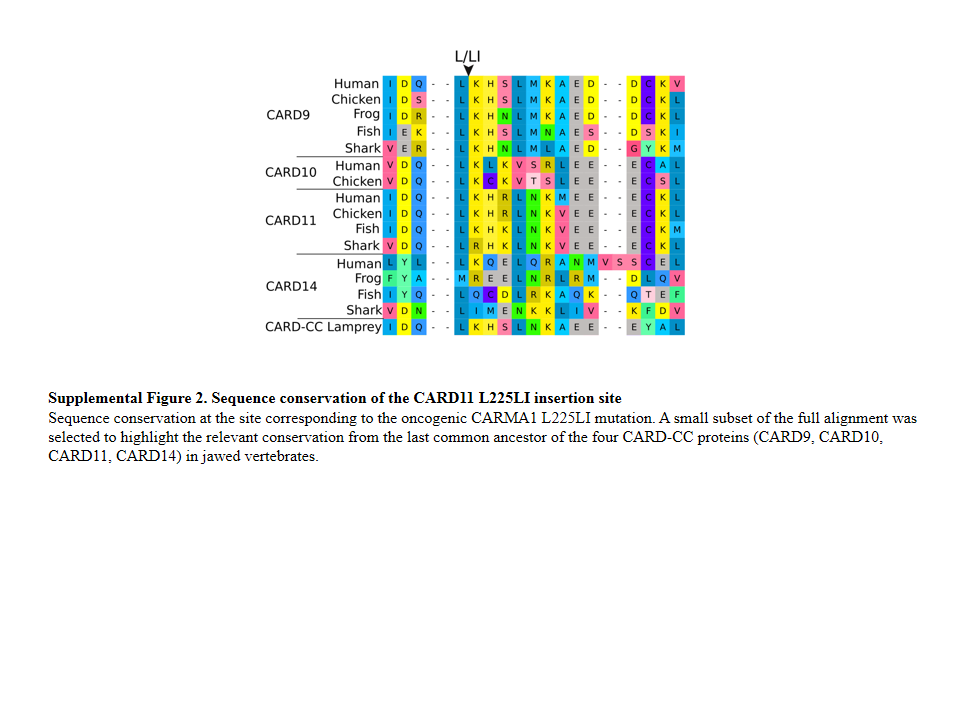

Supplement: Supplementary file 3 [file Image_2.TIF]

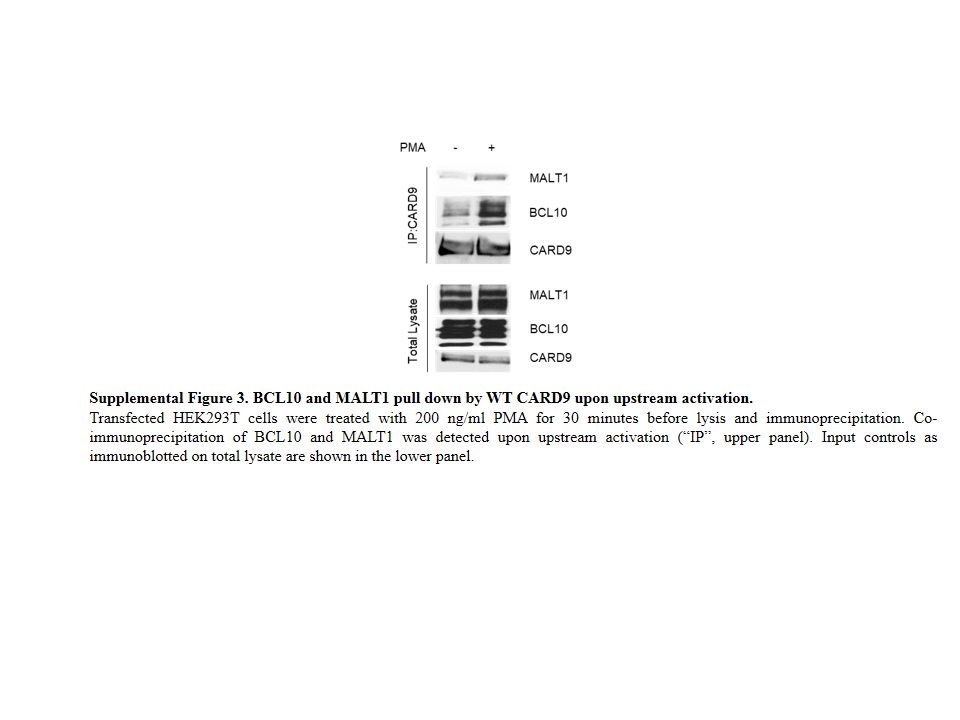

Supplement: Supplementary file 4 [file Image_3.TIF]

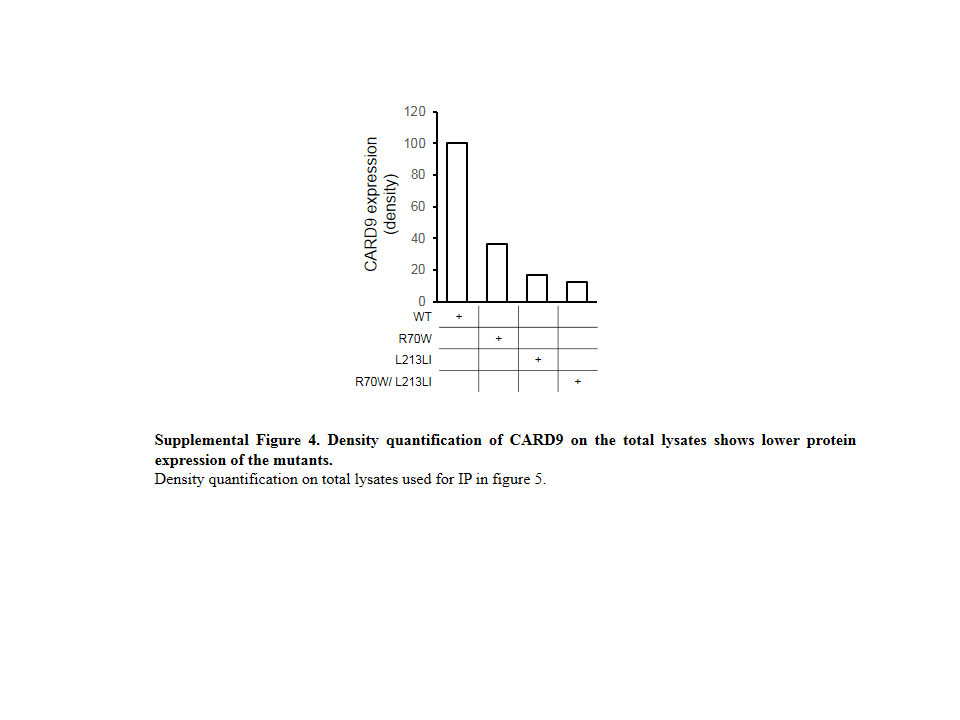

Supplement: Supplementary file 5 [file Image_4.TIF]

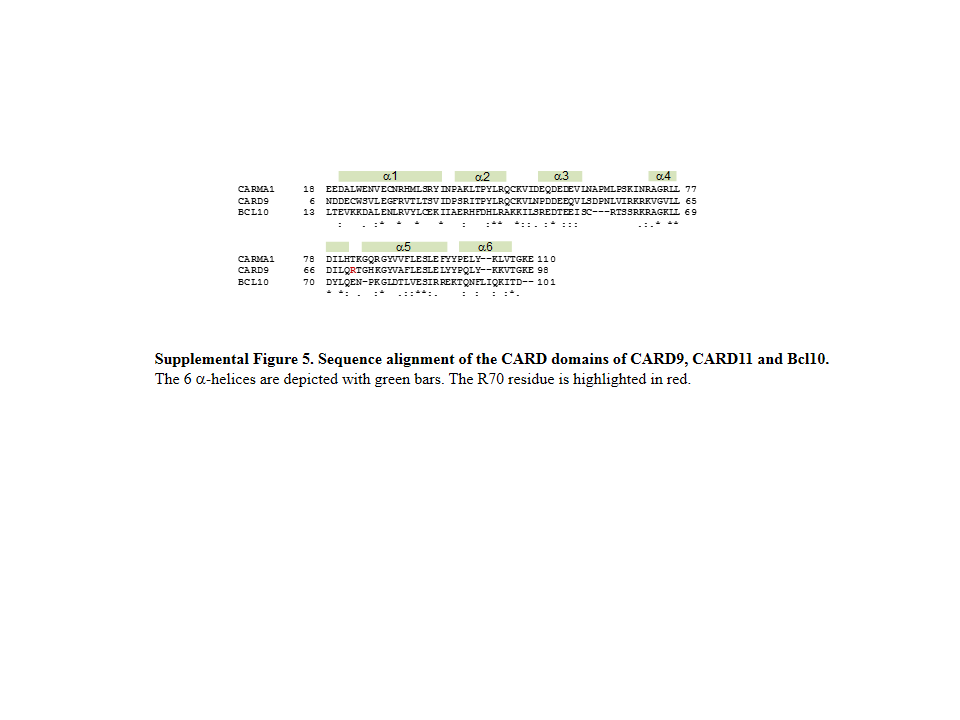

Supplement: Supplementary file 6 [file Image_5.TIF]
